# Evidence for Polyphyly of the Genus *Scrupocellaria* (Bryozoa: Candidae) Based on a Phylogenetic Analysis of Morphological Characters

**DOI:** 10.1371/journal.pone.0095296

**Published:** 2014-04-18

**Authors:** Leandro M. Vieira, Mary E. Spencer Jones, Judith E. Winston, Alvaro E. Migotto, Antonio C. Marques

**Affiliations:** 1 Centro de Biologia Marinha, Universidade de São Paulo, São Sebastião, São Paulo, Brazil; 2 Department of Life Sciences, Natural History Museum, London, United Kingdom; 3 Virginia Museum of Natural History, Martinsville, Virginia, United States of America; 4 Departamento de Zoologia, Instituto de Biociências, Universidade de São Paulo, São Paulo, Brazil; Australian Museum, Australia

## Abstract

The bryozoan genus *Scrupocellaria* comprises about 80 species in the family Candidae. We propose a hypothesis for the phylogenetic relationships among species assigned to *Scrupocellaria* to serve as framework for a phylogenetic classification using 35 morphological characters. Our results suggest that the genus *Scrupocellaria* is polyphyletic. *Scrupocellaria s. str.* is redefined according to four morphological features: vibracular chamber with a curved setal groove, ooecium with a single ectooecial fenestra, two axillary vibracula, and a membranous operculum with a distinct distal rim. Thus, the genus includes only 11 species: *Scrupocellaria aegeensis*, *Scrupocellaria delilii*, *Scrupocellaria harmeri*, *Scrupocellaria incurvata*, *Scrupocellaria inermis*, *Scrupocellaria intermedia*, *Scrupocellaria jullieni*, *Scrupocellaria minuta*, *Scrupocellaria puelcha*, *Scrupocellaria scrupea*, and *Scrupocellaria scruposa*. The monophyly of *Cradoscrupocellaria* is supported and five new genera are erected: *Aquiloniella* n. gen., *Aspiscellaria* n. gen., *Paralicornia* n. gen., *Pomocellaria* n. gen. and *Scrupocaberea* n. gen. Two other new genera, *Bathycellaria* n. gen. and *Sinocellaria* n. gen., are erected to accommodate two poorly known species, *Scrupocellaria profundis* Osburn and *Scrupocellaria uniseriata* Liu, respectively. *Scrupocellaria congesta* is tentatively assigned to *Tricellaria*. Fifteen species are reassigned to *Licornia*: *Licornia cookie* n. comb., *Licornia micheli* n. comb., *Licornia milneri* n. comb., *Licornia curvata* n. comb., *Licornia diegensis* n. comb., *Licornia drachi* n. comb., *Licornia mexicana* n. comb., *Licornia pugnax* n. comb., *Licornia raigadensis* n. comb., *Licornia regularis* n. comb., *Licornia resseri* n. comb., *Licornia securifera* n. comb., *Licornia spinigera* n. comb., *Licornia tridentata* n. comb., and *Licornia wasinensis* n. comb. *Notoplites americanus* n. name is proposed as a replacement name for *Scrupocellaria clausa* Canu & Bassler. Three fossil species are reassigned to *Canda*: *Canda rathbuni* n. comb., *Canda triangulata* n. comb. and *Canda williardi* n. comb. A species is reassigned to *Notoplites*, *Notoplites elegantissima* n. comb. The generic assignment of eleven species of *Scrupocellaria*, including *Scrupocellaria macandrei*, remains uncertain.

## Introduction

The bryozoan genus *Scrupocellaria* van Beneden, 1845 [1], as traditionally understood, comprises about 80 species in the family Candidae d'Orbigny, 1851 [2], [3]. It has been widely reported in shallow marine environments, from tropical to polar areas [4]–[11]. A few species have been reported in deeper water [12]–[14], with greatest recorded depth about 2,000 meters [13]. Species of *Scrupocellaria* have also been reported on artificial substrates [15]–[17]. A few species have been considered exotic and introduced for different localities [18]–[21], but a reassessment of their introduction status and a refined taxonomy suggest the taxa named may be part of species complexes [22] or belong to different genera [23], [24].

The genus *Scrupocellaria* was erected to include *Sertularia scruposa* Linnaeus, 1758 [1]. Later authors added more species and described new characters [25]; consequently, the bryozoan genus *Scrupocellaria* van Beneden, 1845 grew in size and morphological diversity over time. In its broad sense [5], [26], *Scrupocellaria* has been defined as having the following characteristics: erect, biserial, branching colonies anchored to the substratum by rhizoids; rhombic autozooids with partially membranous frontal walls, with spines, including a modified lateral spine (the scutum); zooid polymorphs often including lateral and/or frontal avicularia, almost always including baso-lateral vibracula, and subglobular hyperstomial ooecia. At least three genera were synonymized under *Scrupocellaria*, *viz. Cellarina* van Beneden, 1848 [27], *Crisina* van Beneden, 1850 [28], and *Licornia* van Beneden, 1850 [26], [28]. One of the previously synonymized genera, *Licornia*, has since been treated as a distinct taxon and its generic status restored [23]. Recently, Vieira *et al.* [24] used some morphological features to erect a new genus, *Cradoscrupocellaria*, for some additional species previously assigned to *Scrupocellaria* and described 18 new species.

Neither a morphological nor a molecular phylogenetic hypothesis has been published for any taxa of the family Candidae, despite the molecular evidence for non-monophyletic status of *Scrupocellaria* [29], [30]. Thus, the goals of this study were (i) to assess and provide data on the comparative morphology of *Scrupocellaria sensu lato*, finding previously unrecognized homologies of the character states in order to (ii) propose a hypothesis of the phylogenetic relationships among *Scrupocellaria* to (iii) serve as framework for a phylogenetic classification of the group and the validation of new nomenclatural decisions.

## Materials and Methods

### Nomenclatural Acts

The electronic edition of this article conforms to the requirements of the amended International Code of Zoological Nomenclature, and hence the new names contained herein are available under that Code from the electronic edition of this article. This published work and the nomenclatural acts it contains have been registered in ZooBank, the online registration system for the ICZN. The ZooBank LSIDs (Life Science Identifiers) can be resolved and the associated information viewed through any standard web browser by appending the LSID to the prefix “http://zoobank.org/”. The LSID for this publication is: urn:lsid:zoobank.org:pub:8A2439E2-4B08-419C-BDA2-804A675F8B7B. The electronic edition of this work was published in a journal with an ISSN, and has been archived and is available from the following digital repositories: PubMed Central and LOCKSS.

### Specimens examined

For morphologic phylogenetic analysis 85 species of Candidae were included (Text S1). To prevent incompleteness of data and mislead of the characters/character states in the matrix, we did not include in the phylogenetic analysis: fossil species, species with bad preserved type material, and poorly known species whose type specimens have not been found or examined using scanning electron microscopy. In the absence of any previous phylogenetic hypothesis related to the family Candidae, we selected 8 species from four genera to serve as outgroups for the analysis, *viz. Notoplites* Harmer, 1923, *Tricellaria* Fleming, 1828, *Canda* Lamouroux, 1816 and *Caberea* Lamouroux, 1816. Incorporating these taxa was also important in order to test the monophyly of *Scrupocellaria*. We chose *Notoplites marsupiatus* (Jullien, 1882) to root the unrooted cladogram of the analysis because of its distinct scutum shape (when compared with other Candidae species) and the presence of abfrontal avicularia rather than abfrontal vibracula (a vibracular chamber is considered to be a defining characteristic of *Scrupocellaria* species [5], [11], [26], [31]).

Examined specimens (Recent and Fossil) are deposited in the following collections:

AMNH, American Museum of Natural History (USA)

LSL, Linnean Society of London (United Kingdom)

MCZ, Museum of Comparative Zoology, Harvard University (USA)

MM, Manchester Museum (United Kingdom)

MNHN, Muséum national d'Histoire naturelle (France)

MOM, Musée océanographique de Monaco (Monaco)

MTQ, Museum of Tropical Queensland (Australia)

MZUSP, Museu de Zoologia da Universidade de São Paulo (Brazil)

NMV, Museum Victoria (Australia)

NHMUK, Natural History Museum, London (United Kingdom)

NCB, Nederlands Centrum voor Biodiversiteit Naturalis (Nationaal Natuurhistorisch Museum, Leiden; Netherlands)

SBMNH, Santa Barbara Museum of Natural History (USA)

USNM, National Museum of Natural History, Smithsonian Institution (USA)

VMNH, Virginia Museum of Natural History (USA)

All necessary permits were obtained for the described field studies in Brazil (collecting permit numbers 10186 and 19936 SISBIO/Instituto Chico Mendes de Conservação da Biodiversidade). The reported localities do not include protected areas and did not involve endangered or protected species. Permissions from all museums were obtained to access and study their collections.

### Morphology and phylogenetic analysis

All specimens were examined under the stereomicroscope. Selected specimens were mounted for examination in a scanning electron microscope (SEM) (Zeiss EVO-60, Zeiss LEO 1455-VP and Zeiss DSM 940) for description and selection of the characters. We scored 35 characters for all terminal taxa; some of these characters show homoplasies among the family members (*e.g.* absence of scutum and rhizoids with hooks). All characters were treated as unordered and equally weighted. Autapomorphies of terminal taxa, which do not provide evidence to support monophyly at supraspecific levels, were excluded from the analysis. Unknown states were indicated as “?” in the data matrix, inapplicable states were indicated as (“-”). Whenever necessary, polymorphisms were explicitly considered in the coding and are marked in the data matrix. The list and discussion of characters used are given below.

The character matrix (Table S1) was edited using Mesquite v2.75 [32]. Maximum parsimony analyses were carried out using TNT v1.1 [33], adopting “New Technology” search algorithms (sectorial search, ratchet, tree drifting, and tree fusion) for 3,000 random addition sequences, 10 random number seeds, opting for collapsing trees after the search. The resulting forest of trees was summarized in a semi-strict consensus [34] topology. Bremer support [35] was calculated using the script Bremer.run in TNT, with configuration “search for trees 10 times longer”, “do 10 ratchet iterations in constrained searches”, and other settings following the standard script. The length (L), consistency index (CI) and retention index (RI) for both tree and characters were calculated in TNT. Morphological characters were optimized in the semi-strict consensus tree, and the list of state optimizations was included in supporting information Text S2.

### List of characters used for phylogenetic analysis

#### Rhizoids (Figures 1A–F)

**Figure 1:**
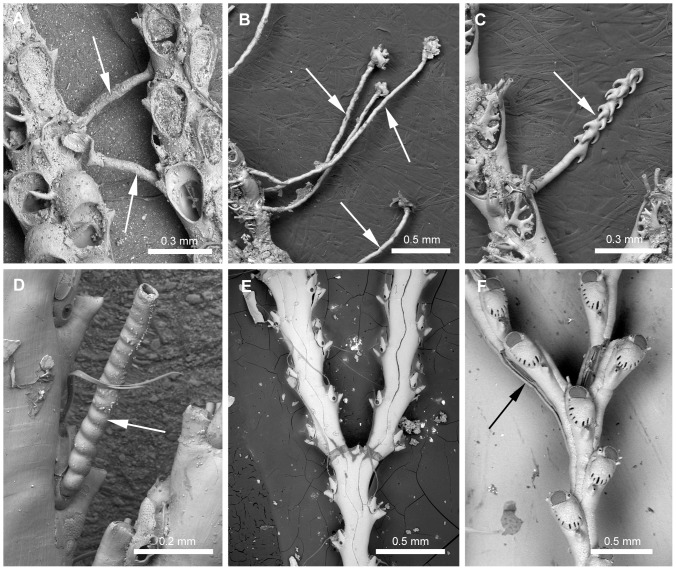
Rhizoids found in Candidae species. Rhizoids found in Candidae species (arrows). A, NHMUK 1926.9.6.84, *Licornia jolloisii* (Audouin, 1826); interconnective rhizoids (white arrow). B–D, Holdfast rhizoids (white arrows). B, NHMUK 1963.3.6.7a, *Cradoscrupocellaria ellisi* (Vieira & Spencer Jones, 2012); smooth rhizoids. C, NHMUK 2010.12.6.1, *Cradoscrupocellaria arisaigensis* Vieira, Spencer Jones & Winston, 2013; rhizoids with retroussé hooks. D, NHMUK 1899.6.1.340, *Licornia cyclostoma* (Busk, 1852); ringed rhizoids. E, USNM 8426, *Paralicornia sinuosa* (Canu & Bassler, 1927) n. comb.; colony without rhizoid on abfrontal surface. F, NHMUK 1887.12.9.83, *Notoplites clausus* (Busk, 1884); colony with adjacent rhizoids on abfrontal surface (black arrow).

**Figure 2:**
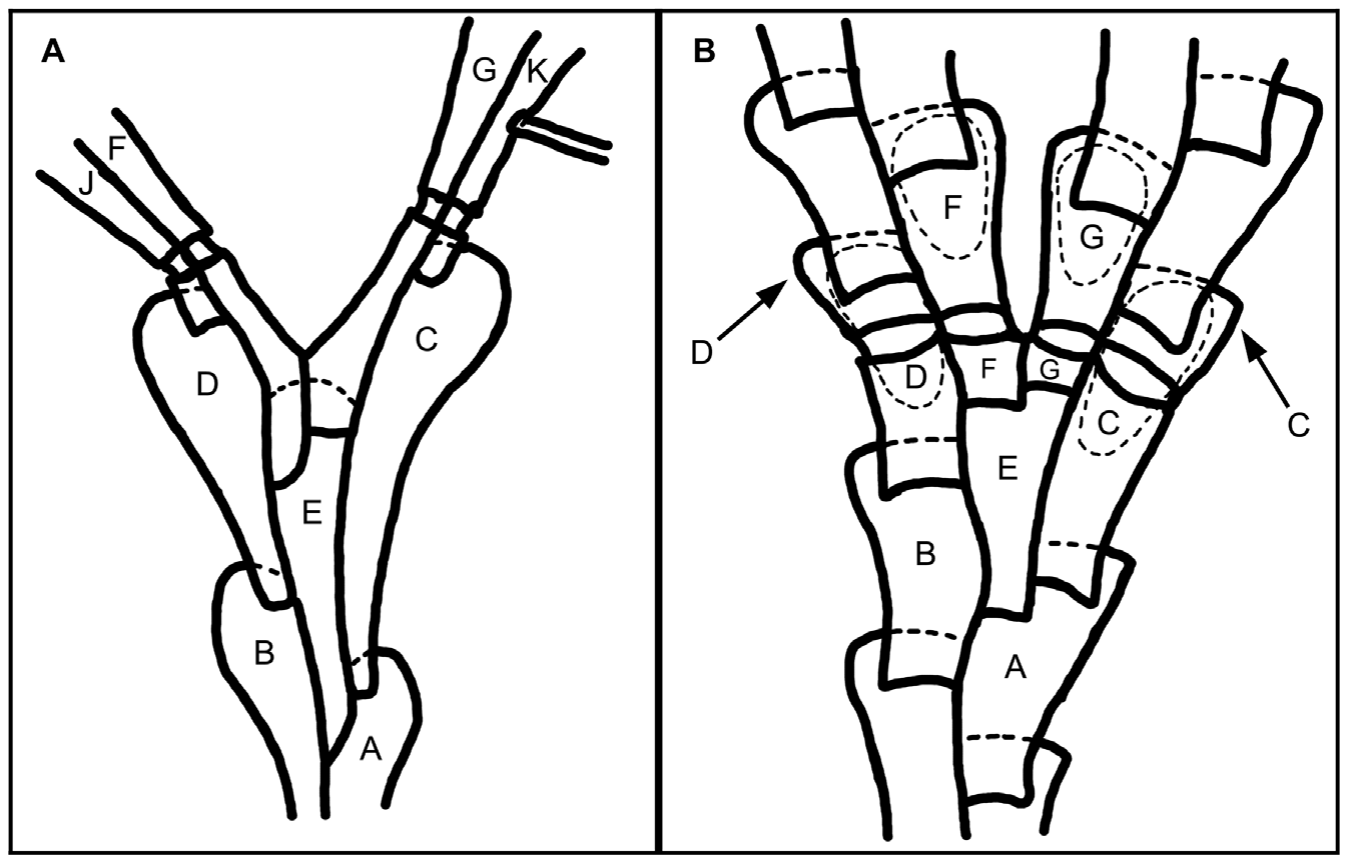
Notation for the order of zooids at branch bifurcations. Abfrontal surface of colony showing the uniform notation for the order of zooids at branch bifurcations proposed by Harmer [25] and the position of the joints (modified from Harmer [25]). A, Joints passing across zooids FD and GC (type 15). B, Joints passing across zooids FJ and GK (type 8).

1. *Transverse tubes connecting adjacent branches* (L = 2; CI = 0.500; RI = 0.833): (0) absent, (1) present (Figure 1A). Remarks. The presence of this character is typical of the species assigned to the genus *Canda* [26] and some species of *Licornia* [23]. The interconnective rhizoid [36] (Figure 1A) arises from a proximal rhizoidal pore of the vibracular chamber and attaches to the pore of the vibracular chamber in the adjacent branch or, rarely, to the abfrontal surface of the zooid in the adjacent branch. Such interconnective rhizoids are quite distinct in function from the holdfast rhizoids that attach to the substrate, that are often found in Candidae species (Figures 1B–D). In *Licornia*, smaller (presumably younger) colonies may not have the connecting tubes, but they are present in more developed colonies (L.M. Vieira, unpubl. data).
2. *Rhizoid surface* (L = 10; CI = 0.200; RI = 0.600): (0) smooth (Figure 1B), (1) with retroussé hooks (Figure 1C), (2) ringed (Figure 1D). Remarks. Hooked rhizoids have been considered a phenotypical variation among *Scrupocellaria* species [26]. This character, however, distinguishes some species of Candidae [23], [24], [37].
3. *Rhizoids adjacent to the abfrontal surface of the colony* (L = 1; CI = 1.000; RI = 1.000): (0) absent (Figure 1E), (1) present (Figure 1F). Remarks. Character present in some species of Candidae, *e.g. Notoplites clausus* and *Notoplites marsupiatus* [26].

#### Branch (Figures 2A–B, 3A–C)

**Figure 3:**
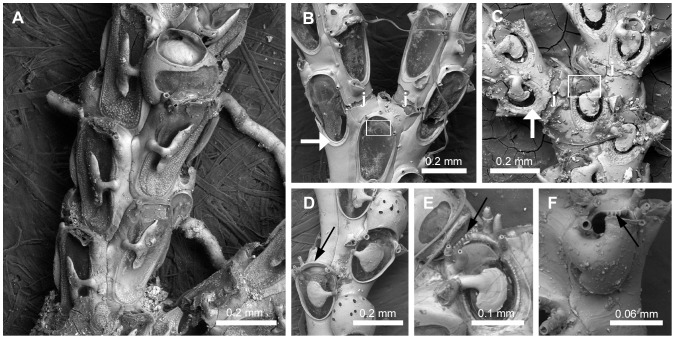
Branch and zooid morphologies. A, NHMUK 1890.1.30.25-27, *Canda retiformis* (Pourtalès, 1867); adjacent zooids sharply inclined in relation to the axis, characteristic of *Canda*. B–C, Position of the joints in zooids C and D at bifurcation; note the development of cryptocyst (white arrows) and two distinct opercula (rectangle). B, NHMUK 1936.12.30.146, *Licornia jolloisii* (Audouin, 1826); the joints (j) pass across the opesiae in zooids C and D; note the membranous operculum with its distinctly chitinous distal rim. C, NHMUK 1899.7.1.780, *Scrupocaberea maderensis* (Busk, 1860) n. comb.; the joints (j) are passing across the gymnocysts in C and D zooids; note the entirely chitinous operculum placed in an obliquely truncate distal area. D–F, Distal edge of autozooid (black arrow). D, NHMUK 1987.1.18.41, *Paralicornia limatula* (Hayward, 1988) n. comb.; smooth distal edge. E, NHMUK 1996.4.26.5, *Scrupocaberea* sp.; corrugated distal edge in ovicelled zooids. F, NHMUK 1934.10.8.1, *Scrupocellaria minuta* (Kirkpatrick, 1888); corrugated distal edge in non-ovicelled zooids.

4. *Position of the joints at bifurcation* (L = 2; CI = 0.500; RI = 0.667): (0) passing across zooids FD and GC (Figure 2B), (1) passing across zooids FJ and GK (Figure 2A). Remarks. Chitinous joints between branches are often reported in Candidae species, maybe as a result of branch fragmentation [26]. The feature is conspicuous in some genera (*Licornia*, *Tricellaria, Notoplites*, and in most species of Candidae). In *Canda* spp., the joints are often seen in older branches; and in a few *Licornia* species (*viz.*, *Licornia curvata*, *Licornia diegensis*, *Licornia drachi*, *Licornia regularis,* and *Licornia securifera*) the joints are inconspicuous due to heavier calcification in all parts of the colonies of those species. The bifurcation pattern of the colony and position of the joints have been adopted to differentiate some genera of branching, erect bryozoans with membranous frontal walls [25], [38], [39]. The notation system used here for the ordering of the zooecia at a branching event is based on the bifurcation of biserial colonies (Figures 2A,B): “A” and “B”, for the two most proximal zooids which form the bifurcation; “C” and “D”, those placed on the outer sides of the branches right before the bifurcation, budding off from “A” and “B” respectively; “E”, the axillary zooid, derived from “A” and lying on the inner side of “C”; “F” and “G”, on the inner side of the branches right after the bifurcation and adjacent to zooids “D” and “C” respectively; “J” and “K”, the zooids derived from “D” and “C”, respectively [25]. This character is not known for *Aspiscellaria bellula* because of the absence of bifurcations in the colonies studied; the joints are apparently rare, present in a single zooid at the base of the colony [40].
5. *Position of the joints in relation to zooids C and D* (L = 5; CI = 0.200; RI = 0.889): (0) passing across the gymnocysts (Figure 3C), (1) passing across the opesiae (Figure 3B).
6. *Position of the joints in relation to zooids J and K* (L = 1; CI = 1.000; RI = 1.000): (0) passing across zooid gymnocysts, (1) passing across the zooid opesiae.
7. *Adjacent zooids along the axis* (L = 1; CI = 1.000; RI = 1.000): (0) placed side by side in the same plane or slightly inclined in relation to the axis (Figures 3B,C), (1) are abruptly inclined, about 250 degrees or more in relation to the axis (Figure 3A). Remarks. *Licornia diadema*, *Cradoscrupocellaria bertholletii*, *Cradoscrupocellaria macrorhyncha,* and *Cradoscrupocellaria reptans* have the frontal surface of adjacent zooids slightly inclined in relation to the axis in apical region of the colony, but placed side by side at the same plane in some branches at the basal region (coded with “0”).

#### Zooid morphology (Figures 3B–F)

8. *Aperture area* (L = 3; CI = 0.333; RI = 0.714): (0) continuous and in the same plane as the frontal membrane (Figure 3B), (1) placed in an obliquely truncate distal area and separated from the frontal membranous area by two suborificial condyles (Figures 3C,E). Remarks. In *Scrupocaberea ornithorhynchus*, the truncate distal area is reduced and shorter than those of *Scrupocaberea dongolensis*, *Scrupocaberea gilbertensis,* and *Scrupocaberea maderensis*.
9. *Distal edge of autozooid* (L = 2; CI = 1.000; RI = 1.000): (0) smooth (Figure 3D), (1) toothed only in ovicelled zooids (Figure 3E), (2) toothed in ovicelled and non-ovicelled zooids (Figure 3F). Remarks. A toothed distal edge in autozooids has not yet been described for Candidae species, because it can only be observed using scanning electron microscopy. The character is considered unknown (‘?’) for *Scrupocellaria harmeri*, whose type is embedded in a Canada balsam preparation.
10. *Morphology of operculum* (L = 3; CI = 0.333; RI = 0.714): (0) membranous and only rim distinctly chitinous (Figure 3B), (1) operculum wholly chitinous (Figure 3C). Remarks. The majority of Candidae species has a membranous operculum continuous with the frontal membrane, but distinguished from it by its inverted-U-shaped and slightly chitinous distal edge. The thick and entirely chitinous opercula of *Notoplites* spp., *Scrupocaberea dongolensis*, *Scrupocaberea gilbertensis*, *Scrupocaberea maderensis,* and *Scrupocaberea ornithorhynchus* are placed in the obliquely truncate distal area.
11. *Cryptocyst* (L = 12; CI = 0.077; RI = 0.368): (0) vestigial, as a very tiny rim around the opesia (Figure 3B), (1) forming a conspicuous stripe around the opesia (Figure 3C).

#### Spines (Figures 1F, 4A–H)

In Candidae the oral spine is characterized by the presence of a jointed base (sometimes with external calcification and distinct from the distal zooecial projections found in *Bugula* species [41]). Candidae species have a variable number of oral spines (0–7 distal spines), some of those may be distinguished by their position at the distal margin of the opesia, *viz.* inner, outer and median spines. Both the presence of polymorphic data and the lack of topographic correspondence suggest that not all spines are homologous, but may be a product of serial homology. Hence, it is only possible to homologize the states between the spines of the same nature, as indicated by topographical correspondence. For example, the most proximal outer spines of different taxa are comparable with each other but not with the proximal inner spines of different taxa or even those in the same individual. Thus, we coded them as three separate characters (Characters 12 and 13 and 14), *i.e.* proximal-most outer, the proximal-most inner and distal-most oral spines.

**Figure 4:**
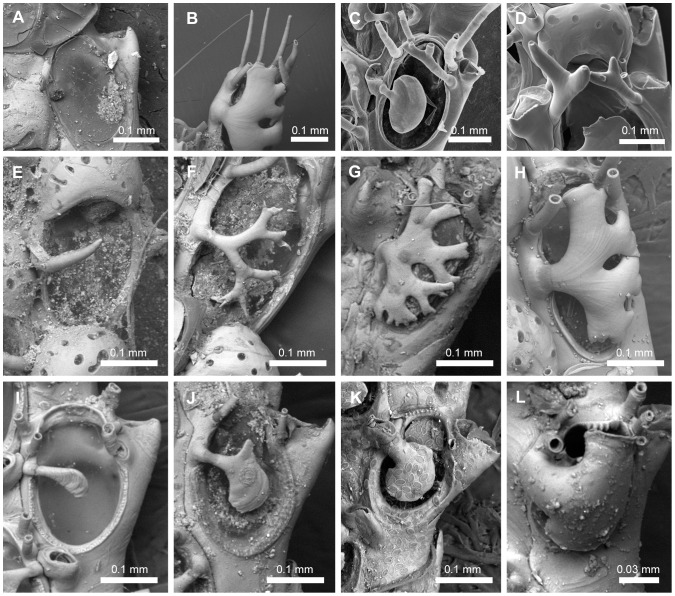
Oral spines and scutum. Oral spines and scutum. A, NHMUK 1911.10.1.367, *Scrupocellaria inermis* Norman, 1867; *z*ooid without distal spines and scutum. B, NHMUK 1882.5.24.8-12, *Cradoscrupocellaria gautieri* Vieira, Spencer Jones & Winston, 2013; zooid with unbranched oral spines. C, MZUSP 266, *Aspiscellaria* sp.; zooid with proximal-most outer spine branched (cervicorn) and additional unbranched spines; note the single ovoid plate of scutum. D, MZUSP 532, *Licornia drachi* (Marcus, 1955) n. comb.; zooid with proximal-outer, outer and inner spines bifurcated, and distal unbranched spines. E–L, Distinct morphologies of scutum at the inner edge of opesia, arising at the median region of opesia—Character 16: E, state 0, NHMUK 1936.12.30.146, *Licornia jolloisii* (Audouin, 1826); F, state 1, NHMUK 2010.10.6.1, *Cradoscrupocellaria tenuirostris* (Osburn., 1950); G, state 2, NHMUK 1911.10.1.353, *Cradoscrupocellaria ellisi* (Vieira & Spencer Jones, 2012); H, state 3, NHMUK 2010.12.6.22, *Cradoscrupocellaria odonoghuei* Vieira, Spencer Jones & Winston, 2013—or from its distal third—Character 17: I–J, state 0 (I, NHMUK 1968.1.18.110, *Pomocellaria californica* (Trask, 1857); J, NHMUK 1911.10.1.373, *Scrupocellaria delilii* (Audouin, 1826)); K, state 2, NHMUK 1928.9.13.98, *Scrupocaberea dongolensis* (Waters 1908); L, state 3, NHMUK 1934.10.8.1, *Scrupocellaria minuta* (Kirkpatrick, 1888).

**Figure 5:**
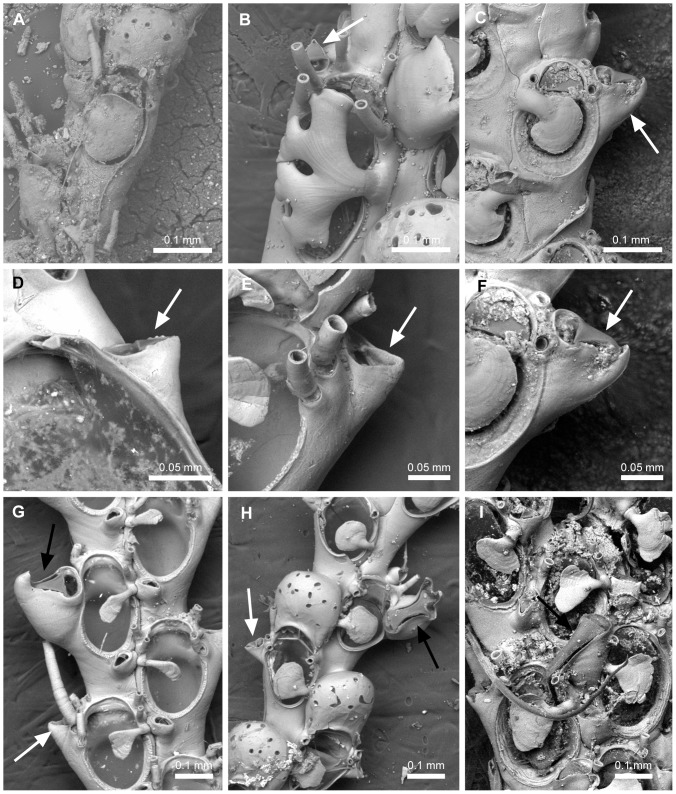
Frontal and lateral avicularia. Frontal and lateral avicularia. A, NHMUK 1888.4.16.200, *Aspiscellaria frondis* (Kirkpatrick, 1890) n. comb.; zooids without lateral avicularium. B–H, Zooids with sessile lateral avicularium (white and black arrows). B, NHMUK 2010.12.6.22, *Cradoscrupocellaria odonoghuei* Vieira, Spencer Jones & Winston, 2013; zooid with avicularium positioned at outer distal corner and directed obliquely distolaterally. C, NHMUK 1899.7.1.804, *Scrupocaberea dongolensis* (Waters 1908) n. comb.; zooid with avicularium positioned lateral to the opercular area and directed laterally. D–F, Rostrum of lateral avicularium. D, NHMUK 1936.12.30.146, *Licornia gaspari* (Thornely 1907); serrated lateral edge, straight at its tips. E, NHMUK 1968.1.18.110, *Pomocellaria californica* (Trask, 1857) n. comb.; smooth lateral edge, with curved tips. F, NHMUK 1899.7.1.804, *Scrupocaberea dongolensis* (Waters 1908) n. comb.; serrated lateral edge, strongly hooked. G–I, Dimorphic lateral and frontal avicularia (black arrows). G, NHMUK 1968.1.18.110, *Pomocellaria californica* (Trask, 1857) n. comb.; dimorphic lateral avicularium with triangular mandible. H, NHMUK 1998.9.29.6, *Paralicornia* sp.; dimorphic lateral avicularium with trifoliate mandible. I, NHMUK 1968.1.18.104, *Licornia diegensis* (Robertson, 1905) n. comb.; dimorphic frontal avicularium.

12. *Proximal-most outer spine* (L = 7; CI = 0.429; RI = 0.556): (0) absent (Figure 4A), (1) present, unbranched (Figure 4B), (2) present, branched in a bifid pattern (Figure 4D), (3) present, branched in a non-bifid pattern (cervicorn) (Figure 4C).
13. *Proximal-most inner spine* (L = 7; CI = 0.429; RI = 0.200): (0) absent (Figure 4A), (1) present, unbranched (Figure 4B), (2) present, branched in a bifid pattern (Figure 4D), (3) present, branched in a non-bifid pattern (cervicorn).
14. *Distal-most spines* (L = 8; CI = 0.250; RI = 0.625): (0) absent (Figure 4A), (1) present, unbranched (Figure 4B), (2) present, branched.
15. *Scutal spine at the inner edge of opesia* (L = 10; CI = 0.200; RI = 0.704): (0) absent (Figure 4A), (1) present, arising at the median region (or slightly below) of the inner part of the opesia (Figures 4C,E–H), (2) present, arising at distal third (Figures 4I–L).
16. *Shape of scutum arising at the median region of opesia* (L = 6; CI = 0.833; RI = 0.957): (0) spine-like and unbranched (Figure 4E), (1) forked to branched, branches homogeneous in width and with sharp tips (Figure 4F), (2) branched and flattened in cross section, branches heterogeneous in width, with a planar frontal surface and truncated tips (Figure 4G), (3) branched and cylindrical in cross section, branches heterogeneous in width, with a convex frontal surface and truncated tips (Figure 4H), (4) forming a single ovoid plate, but with internal channels (visible under light microscope because of their transparency) (Figure 4C), (5) forming an asymmetrical plate, without internal channels. Remarks. *Licornia diadema* has variable shape of scuta, varying from a simple paddle-shaped scutum to an incipient branched scutum bearing one or more slits at the outer margin (coded with “4”).
17. *Shape of scutum arising at the distal third of opesia* (L = 5; CI = 0.800; RI = 0.800): (0) slender base, *i.e.* as wide as distal spines, with unbranched asymmetrical enlarged portion in which the distal region is less developed than the proximal one (Figures 4I–J), (1) slender base, *i.e.* as wide as distal spines, with an irregularly branched enlarged portion, (2) stout base, *i.e.* two or more times wider than distal spines, enlarged portion developed proximally (Figure 4K), (3) stout base, *i.e.* two or more times wider than distal spines, enlarged portion more developed distally than proximally (Figure 4L), (4) stout base, *i.e.* two or more times wider than distal spines, enlarged portion continuous with edge of opesia and with some slits at the outer margin (Figure 1F).
18. *Development of the unbranched asymmetrical portion of the scutum at the distal third of opesia* (L = 2; CI = 0.500; RI = 0.800): (0) narrow paddle-shaped, curved towards the proximal region of opesia (Figure 4I), (1) enlarged shield-shaped, not curved towards the proximal region of opesia (Figure 4J).

#### Frontal and lateral avicularia (Figures 5A–F)

19. *Sessile distolateral avicularium* (L = 5; CI = 0.200; RI = 0.714): (0) absent (Figure 5A), (1) present (Figures 5B–H).
20. *Direction of rostrum of sessile lateral avicularium* (L = 3; CI = 0.667; RI = 0.889): (0) lateral (Figure 5C), (1) obliquely laterodistal (Figure 5B), (2) obliquely lateroproximal. Remarks. In species with a sessile laterodistally directed avicularium the avicularium is often obscured by the outer distal spines, therefore more difficult to see.
21. *Rostrum of lateral avicularium* (L = 4; CI = 0.500; RI = 0.900): (0) serrated lateral edge, straight to slightly curved at its tips (Figure 5D), (1) smooth lateral edge, with curved tips (Figure 5E), (2) serrated lateral edge, strongly hooked (Figure 5F).
22. *Giant lateral avicularium* (L = 4; CI = 0.500; RI = 0.667): (0) absent, (1) present, with triangular to elongate mandible (Figure 5G), (2) present, with trifoliate mandible (Figure 5H).
23. *Dimorphic frontal avicularium* (L = 12; CI = 0.250; RI = 0.727): (0) absent, (1) present, with triangular mandible (Figure 5I), (2) present, with lanceolate mandible, (3) present, with trifoliate mandible.

#### Ovicells (Figure 6A–C)

**Figure 6:**
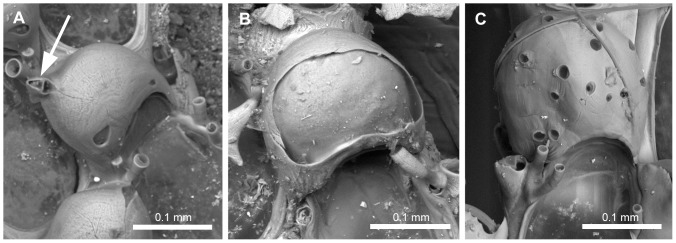
Sessile ooecia found in Candidae. Sessile ooecia found in Candidae. A, NHMUK 1966.1.10.9, *Scrupocellaria scruposa* (Linnaeus, 1758); ooecium with a small drop-shaped pore with a distal avicularium (white arrow). B, NHMUK 1968.1.18.111, *Pomocellaria varians* (Hincks, 1882) n. comb.; ooecium with single, large fenestra, without avicularium. C, NHMUK 1899.7.1.736, *Cradoscrupocellaria bertholletii* (Audouin, 1826); ooecium with porous ectooecium.

24. *Surface of ectooecium* (L = 5; CI = 0.333; RI = 0.867): (0) with a single proximal fenestra (Figure 6A), (1) with a single wide fenestra occupying the majority of the surface of the ooecium (Figure 6B), (2) porous (Figure 6C). Remarks. The single fenestra is reduced to a minute drop-shaped pore in *Scrupocellaria scruposa* (Figure 6A) and one minute pore in *Scrupocellaria aegeensis*. Smooth ooecia were described for *Scrupocellaria delilii* [42], but an uncalcified proximal area, like those of *Tricellaria arctica*, is often seen in the proximal border of the ectooecium (L.M. Vieira, unpubl. data; coded with “0”).
25. *Sessile avicularium associated with ooecium* (L = 1; CI = 1.000; RI = 1.000): (0) absent (Figures 6B,C), (1) present (Figure 6A).

#### Abfrontal heterozooids (Figures 7A–I)

**Figure 7:**
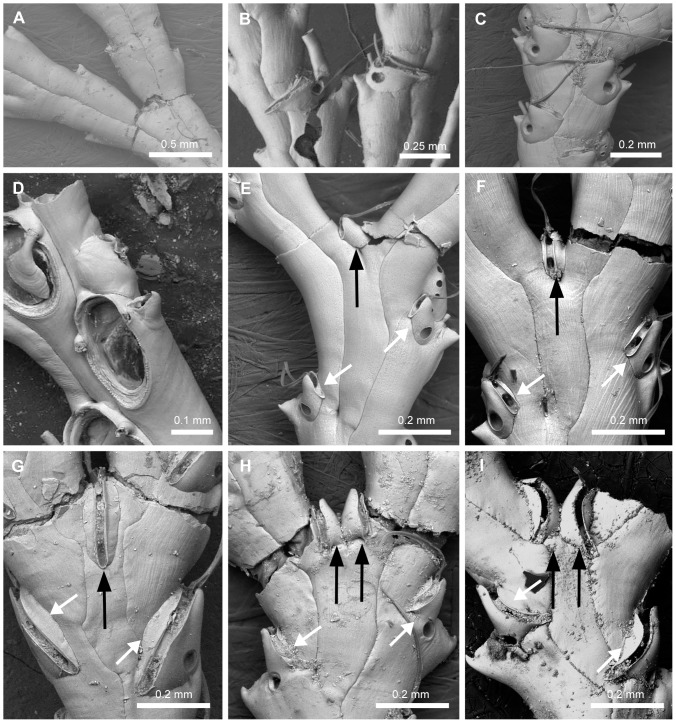
Heterozooids on abfrontal surface of the colony. Heterozooids on abfrontal surface of the colony. A, NHMUK 1911.10.1.385, *Tricellaria congesta* (Norman, 1903); colony without abfrontal heterozooids. B, NHMUK 1911.10.1.376, *Aquiloniella scabra* (van Beneden, 1848) n. comb.; abfrontal avicularia. C, NHMUK 1929.4.26.17, *Cradoscrupocellaria galapagensis* Vieira, Spencer Jones & Winston, 2013; abfrontal vibracula, with transverse setal groove. D, lateral rhizoidal chamber associated with outer zooids at the bifurcation in *Tricellaria* sp.; note the absence of abfrontal avicularia. E–I, Abfrontal vibracula with oblique setal groove (axial vibracula shown by black arrow and other vibracula shown by white arrow). E, NHMUK 1987.1.18.41, *Paralicornia limatula* (Hayward, 1988) n. comb.; vibracula with straight setal groove occupying less than half the length of the vibracular chamber; the setal groove of the axial vibraculum is laterally placed. F, NHMUK 1936.12.30.146, *Licornia gaspari* (Thornely 1907); vibracula with straight setal groove occupying two thirds of the length of the vibracular chamber; the palate is complete and with a small foramen. G, NHMUK 1928.9.13.105, *Licornia curvata* (Harmer, 1926) n. comb.; vibracula with straight setal groove occupying the entire length of the vibracular chamber; note the presence of single axial vibraculum. H, NHMUK 1911.10.1.373, *Scrupocellaria delilii* (Audouin, 1826); vibracula with curved setal groove occupying two thirds of the total length of the vibracular chamber; note the presence of two axial vibracula (black arrows). I, MOM 420193, *Scrupocellaria incurvata* Waters 1897; vibracula with curved setal groove occupying the entire length of the vibracular chamber; note the presence of two axial vibracula (black arrows).

26. *Heterozooids on abfrontal surface of the colony* (L = 3; CI = 0.667; RI = 0.889): (0) absent (Figure 7A), (1) present, as vibracula (Figures 7C,E–I), (2) present, as avicularia (Figure 7B). Remarks. Some Candidae species have abfrontal avicularia, *i.e.* modified zooids that lack a functional polypide and with modified operculum (mandible) [43]–[45]. Two distinct types of avicularia are found on the abfrontal surface of Candidae species: adventitious avicularia, with acute mandibles and one pore in the plane of the palate; and vibracula, with toothed setiform mandibles (setae) and with a tubular orifice at the base of seta [45].
27. *Lateral rhizoidal (rootlets) chamber associated with outer zooids at the bifurcation* (L = 1; CI = 1.000; RI = 1.000): (0) absent, (1) present (Figure 7D). Remarks. The relation between the joints and holdfast rhizoids is not altered during the development of the colony [25]. Species of *Tricellaria* produce holdfast rhizoids on the proximal sides of the joints, while *Notoplites* species produce holdfast rhizoids on the distal side of the joints [25], [26].
28. *Palatal surface of setal groove in vibracular chamber* (L = 4; CI = 0.500; RI = 0.933): (0) present, complete, without foramen (Figures 7C,E), (1) present, complete, with a foramen (opesium) (Figure 7F), (2) absent (Figure 7I). Remarks. In some species the palatal surface is variable, entirely complete to partially incomplete, near the tubular orifice (coded with “0”; Figure 7E).
29. *Position of the setal groove on vibracular chamber (non axial vibracula) in relation to the internodal axis* (L = 1; CI = 1.000; RI = 1.000): (0) transverse (Figure 7C), (1) oblique (Figures 7E–I).
30. *Shape of oblique setal groove in vibracular chamber* (L = 10; CI = 0.500; RI = 0.792): (0) curved, of medium length, occupying at maximum two thirds of the inner margin of the vibracular chamber length; setal groove developed up to half of the width of the zooid (Figure 7H), (1) curved, long, occupying entire inner margin of the vibracular chamber; setal groove developed up to the width of the zooid (Figure 7I), (2) straight, short, occupying half of the length of the vibracular chamber; setal groove placed distally to the rhizoidal pore (Figure 7E), (3) straight, medium, occupying two thirds of the length of the vibracular chamber; setal groove reaching the median part of the rhizoidal pore (Figure 7F), (4) straight, long, occupying the entire length of the vibracular chamber; setal groove passing through the lateral region of the rhizoidal pore but not reaching the line defined by the juxtaposed lateral walls of the zooids (Figure 7G), (5) straight, very long, greater than the length of the vibracular chamber and reaching the line defined by the juxtaposed lateral walls of the zooids.
31. *Number of axial vibracula* (L = 2; CI = 0.500; RI = 0.929): (0) one (Figures 7C,E–G), (1) two (Figures 7H–I).
32. *Setal groove in a single axial vibraculum* (L = 1; CI = 1.000; RI = 1.000): (0) lateral (Figure 7E), (1) longitudinal (Figures 7F,G).
33. *Setal morphology* (L = 7; CI = 0.125; RI = 0.300): (0) delicate, translucent white, (1) hard, chitinous, yellowish-gold.
34. *Surface of setae* (L = 1; CI = 1.000; RI = 1.000): (0) smooth, (1) barbate.
35. *Length of setae* (L = 2; CI = 0.500; RI = 0.929): (0) short, as long as internode width, shorter than zooid length (Figure 7H), (1) long, longer than internode width, longer than zooid length (Figure 7C).

## Results and Discussion

### Phylogenetic analysis

**Figure 8:**
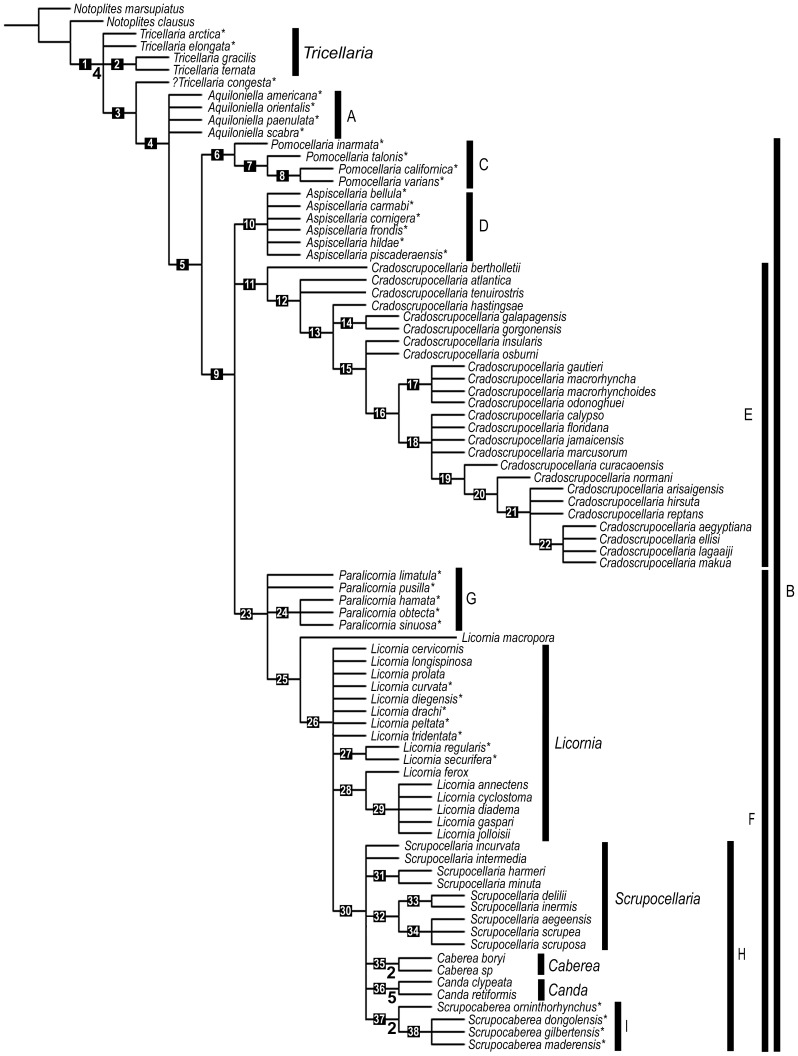
Semi-strict consensus tree. Semi-strict consensus tree based on *Notoplites* as outgroup. New combinations are marked with an asterisk (*). Values below branches refer to Bremer supports (>1).

The TNT analysis yielded 49 most parsimonious trees (L = 126; CI = 0.492; RI = 0.867). The semi-strict consensus tree (L = 156; CI = 0.397; RI = 0.805) shows a polyphyly of the genus *Scrupocellaria s. l.* (Figure 8). Most clades have very low support values (Bremer support of 1), but Node 36 (*Canda*) has Bremer support of 5; Node 1 has Bremer support of 4; and the Nodes 35 (*Caberea*) and 37 (*Scrupocaberea* n. gen.) have Bremer support of 2 (Figure 8).

#### Tricellaria

The basal part of the tree of the Candidae has a low resolution, probably because of the high number of characters coded as inapplicable in those basal taxa causing unstable relationships. Taxonomically, we decided to consider two species of the basal polytomies to be part of the genus *Tricellaria* Fleming, 1828, *viz. Tricellaria arctica* n. comb. (*Menipea arctica* Busk, 1855, also referred to *Scrupocellaria arctica*) and *Tricellaria elongata* n. comb. (*Cellularia scabra* f. *elongata* Smitt, 1868, also referred to *Scrupocellaria elongata*), instead of creating new generic names to accommodate these species. Our decision is based on some shared characters, such as (i) absence of abfrontal vibracula/avicularia, (ii) presence of a lateral rhizoidal chamber associated with the outer zooids of the bifurcation, (iii) a scutum arising from the distal third of the inner opesial rim and (iv) joints passing across the gymnocyst of outer zooids in the zooids C and D. The species described as *Scrupocellaria congesta* is distinguishable from other *Tricellaria* in having joints passing across the opesia of outer zooids in the zooids C and D (assigned in the tree with question mark). *Tricellaria* is the sister group of *?Tricellaria congesta* (genus *incertae sedis*) + Group A + Clade B.

#### Group A (*Aquiloniella* n. gen.): taxa with basal avicularium

A polytomy of four species is here considered to be part of Group A and a large Clade B (with an unambiguous synapomorphy: presence of abfrontal vibracular chamber). The unresolved polytomy would allow a monophyletic Group A to be tested in future analysis. Nomenclaturally, it is convenient to consider the species of Group A as belonging to a unique genus, described below as *Aquiloniella* n. gen. All species of *Aquiloniella* n. gen. are endemic to Arctic and sub-Arctic waters, and may be characterized by colonies with chitinous joints passing across the gymnocyst of outer zooids of the bifurcation, as do those of *Tricellaria*, but with abfrontal avicularia. Thus, they are distinct from *Tricellaria* in the absence of the lateral rhizoidal chamber associated with the outer zooids of the bifurcation, in the presence of abfrontal avicularia, and a scutum arising at the median region of the inner part of the opesia.

#### Clade B: taxa with abfrontal vibracula

The analysis supports the monophyly of the species of Candidae with an abfrontal vibracular chamber (Clade B). The switch from smooth lateral edge to serrated lateral edge of the rostrum of the lateral avicularium is another synapomorphy for this clade. This clade comprises four monophyletic groups (Clade C + Clade D + Clade E + Clade F). The topology of Clade B shows an interesting history concerning the development of setal groove of the vibracular chamber along the evolution of the species of Candidae. The possession of a vibracular chamber with transversal setal groove would be primitive (plesiomorphic), as it is present in three of the included clades (Clade C, Clade D, and Clade E), and the change to an oblique setal groove would be synapomorphic for Clade F.

#### Clade C (*Pomocellaria* n. gen.): the eastern Pacific clade

Clade C seems to be endemic to the Eastern Pacific. It has a basal phylogenetic position in Clade B and it is characterized by (i) an abfrontal vibracular chamber with a transversal setal groove, (ii) ooecium with a large ectooecial fenestra, and (iii) a single axial vibracula. Presence of abfrontal vibracula with a transverse setal groove is a character also present in Clade D and Clade E, but the two clades are distinguished from Clade C by the presence of an ooecium with some ectooecial pores in Clade D and E. The dimorphic lateral avicularia, characteristics of *Pomocellaria californica* n. comb. and *Pomocellaria varians* n. comb., are absent in *Pomocellaria inarmata* n. comb. and *Pomocellaria talonis* n. comb. In the basalmost taxon, *Pomocellaria inarmata*, the scutum and oral spines are absent.

#### Clade D (*Aspiscellaria* n. gen.) and Clade E (*Cradoscrupocellaria*)

Members of Clade D and Clade E have a circumtropical distribution in shallow waters. Clade D has an unambiguous synapomorphy, the outer spines are branched three or more times (cervicorn); this clade also includes species with an oval scutum with internal channels (ornamentation), whereas the branched scutum with a planar frontal surface, present in *Aspiscellaria bellula* n. comb. and in *Cradoscrupocellaria*, may be considered a homoplasy.

The monophyly of the genus *Cradoscrupocellaria* is supported here (Clade E). This genus is characterized by the presence of (i) articulate distal unbranched spines, (ii) a branched scutum arising from midline of the inner edge of the opesia, (iii) a trapezoidal vibracular chamber, (iv) single axial vibraculum, and (v) an ooecium with some ectooecial pores [24]. Character 16 (the shape of the scutum arising at the median region of opesia) is plesiomorphic for character state 1, found in majority of species, while character state 2 and 3 are apomorphic and have an independent origin.

#### Clade F: taxa with a vibracular chamber with oblique setal groove

Clade F has an unambiguous synapomorphy, a vibracular chamber with an oblique setal groove, derived from a primitive condition of a vibracular chamber with transversal setal groove which becomes a short to long oblique setal groove.

#### Group G (*Paralicornia* n. gen.)

The basal part of the Clade F has a polytomy including one monophyletic clade (*Paralicornia hamata* n. comb. + *Paralicornia obtecta* n. comb. *+ Paralicornia sinuosa* n. comb.) defined by a dimorphic lateral avicularium with trifoliate rostrum, and two other species, *Paralicornia limatula* n. comb. and *Paralicornia pusilla* n. comb. The phylogeny suggests that the polytomy may comprise a distinct clade with circumtropical distribution (Group G, named *Paralicornia* n. gen.) due to the (i) absence of bifurcated oral spines (characteristic of *Licornia* species), (ii) the presence of joints passing across the gymnocysts of outer zooids at the branch, and (iii) the presence of a shorter setal grove than those of *Licornia* species.

#### *Licornia*: a paraphyletic group

The monophyly of *Licornia* is not supported by the analysis and the position of some *Licornia* species remains unresolved, most likely because of the elevated number of polymorphic characters included in the data matrix. At the same time, the diagnostic characteristics of the genus described by Vieira *et al*. [23] are insufficient to distinguish species of *Licornia* from six species previously assigned to *Scrupocellaria*. Thus, we herein transfer these taxa to the genus *Licornia*: *Licornia curvata* (Harmer, 1926) n. comb., *Licornia diegensis* (Robertson, 1905) n. comb., *Licornia drachi* (Marcus, 1955) n. comb., *Licornia regularis* (Osburn, 1940) n. comb., *Licornia securifera* (Busk, 1884) n. comb., and *Licornia tridentata* (Waters, 1918) n. comb. (despite the differences in the length of setal grooves, which are longer than those of *Licornia*, and the presence of barely chitinized setae in *Licornia*). According to the phylogeny, the taxonomic position of *Licornia macropora* remains uncertain.

#### Clade H: polytomy of taxa including *Scrupocellaria s. str*

Despite the low resolution of the semi-strict tree, we use a part of the polytomous taxa of Clade H—characterized by presence of an avicularium at the outer wall of the ooecium—to redefine the genus *Scrupocellaria s. str.* according to four morphological features: (i) vibracular chamber with curved setal groove, (ii) ooecium with a single and small ectooecial fenestra, (iii) two axillary vibracula, and (iv) a membranous operculum with a distinct distal rim. Thus, the genus *Scrupocellaria* encompasses only nine species from the polytomy: *Scrupocellaria harmeri*, *Scrupocellaria minuta*, *Scrupocellaria delilii*, *Scrupocellaria incurvata*, *Scrupocellaria inermis*, *Scrupocellaria intermedia*, *Scrupocellaria scrupea*, *Scrupocellaria scruposa,* and *Scrupocellaria aegeensis*. Although these species do not form a unique group, future studies may test the monophyly of the genus.

#### Clade I (*Scrupocaberea* n. gen.)

The monophyletic Clade I, here described as *Scrupocaberea* n. gen., comprises four species distinct from *Scrupocellaria s. str.* in having a (i) well chitinized operculum placed in an obliquely truncate distal area, and a (ii) scutum with a stout base arising at the distal third of opesia and with an enlarged portion developed proximally. Hence, *Scrupocaberea* has a mixture of the morphological features of *Caberea* (*viz.* scutum with stout base and enlarged portion developed proximally, arising from the distal third of the inner opesial rim; distinct opercular area with strongly chitinous operculum) and *Scrupocellaria* (*viz*. vibracular chamber with obliquely curved setal groove). *Caberea*, however, has been morphologically well defined due to the well-developed vibracular chamber with a very long setal groove and barbate seta [26], [31], [46] and, in the present phylogeny, the monophyly of the genus *Caberea* is supported by these two unambiguous synapomorphies. The genus *Canda*, previously characterized by the presence of rhizoids forming cross connections between branches and zooids in two series with their frontal surfaces facing obliquely outwards from the axis [46], has its monophyly supported by three unambiguous synapomorphies, *viz*. (i) joints passing across opesiae of the zooids J and K at the bifurcation, (ii) adjacent zooids abruptly inclined in relation to the axis, (iii) scutum arising at the median region of opesia and forming an asymmetrical plate, without internal channels.

### Systematic account

#### Genus *Scrupocellaria* van Beneden, 1845 s. str

**Figure 9:**
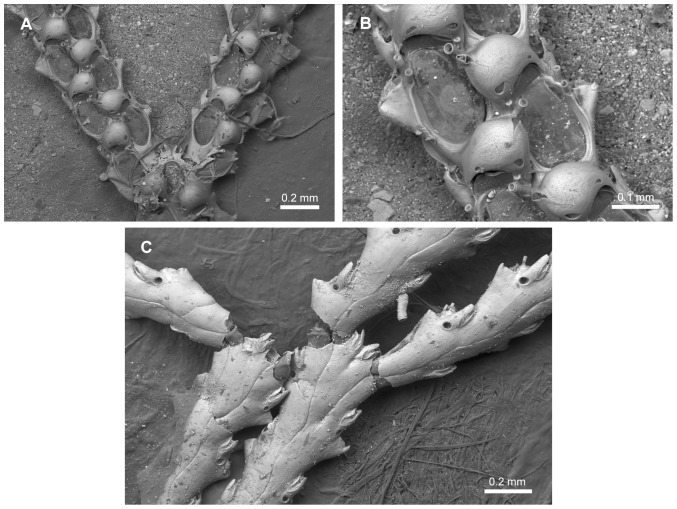
Scanning electron micrographs of *Scrupocellaria scruposa* (Linnaeus, 1758). *Scrupocellaria scruposa* (Linnaeus, 1758). A,C. NHMUK 1966.1.10.9, English Channel, United Kingdom; B. NHMUK 1847.9.15.28, British coast. A, Frontal surface of the bifurcation. B, Close-up of the frontal surface of the ovicelled zooid. C, Abfrontal surface of the colony.

**Figure 10:**
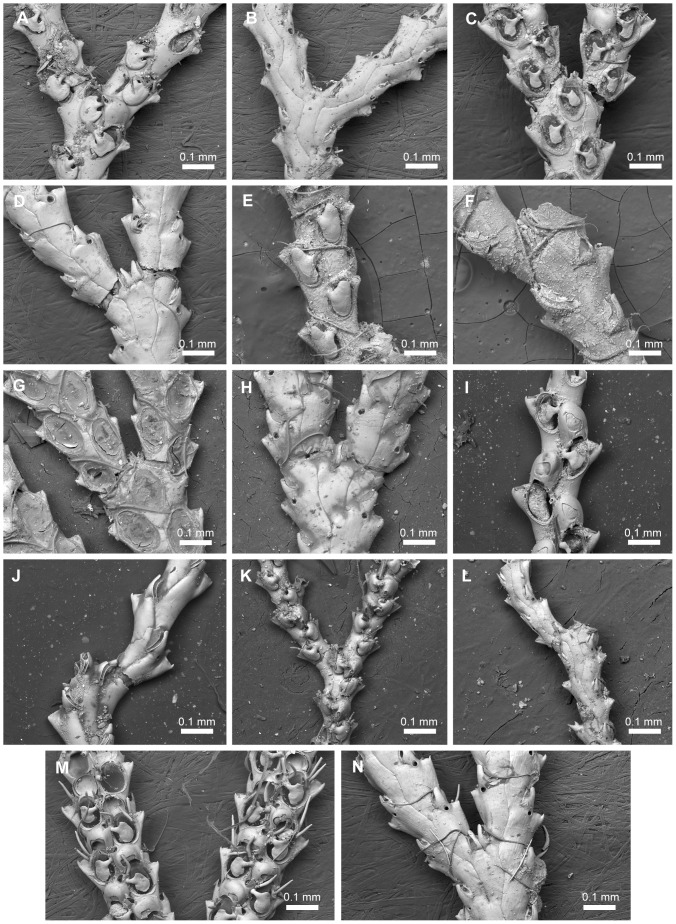
Species assigned to *Scrupocellaria* van Beneden, 1845 *s. str*. Species assigned to *Scrupocellaria* van Beneden, 1845 *s. str*. A–B, *Scrupocellaria aegeensis* Harmelin, 1969, NHMUK 2010.12.7.3, Aegean Sea. C–D, *Scrupocellaria delilii* (Audouin, 1826), NHMUK 1911.10.1.373, Adriatic. E–F, *Scrupocellaria incurvata* Waters, 1897, NHMUK 1899.7.1.797, Crete, Mediterranean. G–H, *Scrupocellaria inermis* Norman 1867, NHMUK 1911.10.1.367, syntype, British coast. I–J, *Scrupocellaria intermedia* Norman, 1896, NHMUK 1911.10.1.369, syntype, Norway. K–L, *Scrupocellaria minuta* (Kirkpatrick, 1888), NHMUK 1934.10.8.1, Mauritius. M–N, *Scrupocellaria scrupea* Busk, 1851, NHMUK 2010.12.8.5, Guernsey.

(Figures 9A–C,10A–N; Text S3)

*Scrupocellaria* van Beneden, 1845: 26 [1].

#### Type species

*Sertularia scruposa* Linnaeus, 1758 (Figures 9A–C), by original designation.

#### Diagnosis

Candidae with jointed branches, almost rectangular zooids, tapering proximally and with broadly oval opesia occupying most of the frontal surface. Joints crossing or slightly below the opesia of outer zooids and crossing the gymnocyst of the inner zooids at the bifurcation. Cryptocyst present or reduced. Oral spines often present, unbranched. Frontal scutum sometimes present, asymmetrical, arising from distal third of the inner margin of the opesia or slightly below it. Lateral avicularia present, aquiline, with a serrated rostrum and hooked tip. Frontal avicularia often present, small, monomorphic. Vibracular chamber almost triangular, with a rhizoidal foramen; setal groove curved and directed obliquely; 2 axillary vibracula. Ooecium with single ectooecial fenestra and a small avicularium at its outer border (Figures 9A–C).

#### Remarks

Tilbrook and Vieira [11] and Vieira *et al.* [23], [24] noted that bryozoan taxonomists have long considered *Scrupocellaria* van Beneden, 1845 to be a well-defined genus, despite the mixtures of characters seen among the many species assigned to it. According to the diagnostic features described above the genus is now redefined to accommodate 11 of the species previously assigned to it: *Sl. aegeensis* Harmelin, 1969 [47] (Figures 10A–B), *Sl. delilli* (Audouin, 1826) [48], [49] (Figures 10C–D), *Sl. harmeri* Osburn, 1947 [40] (the type specimens could not be figured), *Sl. incurvata* Waters, 1897 [38] (Figures 10E–F;  = *Scrupocellaria aquitanica* Jullien & Calvet, 1903 [50]), *Sl. inermis* Norman, 1867 [51] (Figures 10G–H;  = *Scrupocellaria grimaldii* Jullien & Calvet, 1903 [50]), *Sl. intermedia* Norman, 1893 [52] (Figures 10I–J), *Sl. jullieni* Hayward, 1978 [53] (specimens have not been examined using SEM; thus, this species was not included in the phylogeny), *Sl. minuta* Kirkpatrick, 1888 [54], [55] (Figures 10K–L), *Sl. puelcha* (d'Orbigny, 1841) *n. sta.* [56], [57] (specimens have not been examined using SEM; thus, this species was not included in the phylogeny), *Sl. scrupea* Busk, 1851 [58] (Figures 10M–N) and *Sl. scruposa* (Linnaeus, 1758) [59], [60] (Figures 9A–C). The assignment of *Scrupocellaria macandrei* Busk, 1852 [61] to *Scrupocellaria s. str.* was not confirmed because the morphological characteristics of the species cannot be recognized in the type specimen (NHMUK 1854.11.14.78).

Five new genera are erected to accommodate species included in the present phylogeny: *Aquiloniella* n. gen., *Aspiscellaria* n. gen., *Paralicornia* n. gen., *Pomocellaria* n. gen. and *Scrupocaberea* n. gen. (see below). Two other new genera, *Bathycellaria* n. gen. and *Sinocellaria* n. gen. are erected to accommodate two additional species (specimens have not been examined using SEM; thus, they were not included in phylogeny; see Remarks on other species previously assigned to *Scrupocellaria*). The diagnosis of *Licornia* [23] is emended to include species with uncurved setal groove directed obliquely to the axis of the internode and extending two-thirds or more of the vibracular chamber length; thus, in addition to the six species included in the phylogenetic analysis (Figure 8), another eight species are reassigned to *Licornia* (see below).

The curved setal groove of the vibraculum of members of the genus *Scrupocellaria s. str.* resembles those of *Canda* and *Scrupocaberea* n. gen.; the genus *Canda* is distinct in the shape of the internodes, position of the joints in relation to the bifurcation, the presence of interconnective rhizoids, and a well-developed cryptocyst. The genus *Scrupocaberea* n. gen. is distinguishable from *Scrupocellaria s. str.* by the presence of a well-chitinized operculum placed at the distal truncate area of the zooid (see below).

*Scrupocellaria inermis* is distinguishable from the other species of the genus by the absence of oral spines and scutum. The scutum is also absent in the type of the genus, *Sl. scruposa*. *Scrupocellaria aegeensis*, described from Mediterranean, has a scutum with a convex distal edge and cuspidate projections at the proximal and distal inner rim. *Scrupocellaria minuta* has a distinctly different scutum with a stout base three times wider than the distal spines and an enlarged portion more developed distally than proximally. Small differences are seen in scuta shape of *Sl. delilii*, *Sl. harmeri* and *Sl. scrupea*; they can be clearly distinguished by the position of the joints across the bifurcation, and the shape and size of abfrontal vibracula. *Scrupocellaria intermedia* and *Sl. jullieni* are distinct among *Scrupocellaria* species in having a scutum arising from the median region of the inner part of the opesia; *Scrupocellaria intermedia* is also distinct in having dimorphic lateral avicularia with trifoliate rostra. Other species with trifoliate lateral avicularia are assigned to *Paralicornia* n. gen. (see below). *Scrupocellaria incurvata* has a large scutum, as wide as the opesia; this species is also characterized by the presence of dimorphic lateral avicularia with a triangular, laterally directed mandible.

#### Genus Aquiloniella n. gen

**Figure 11:**
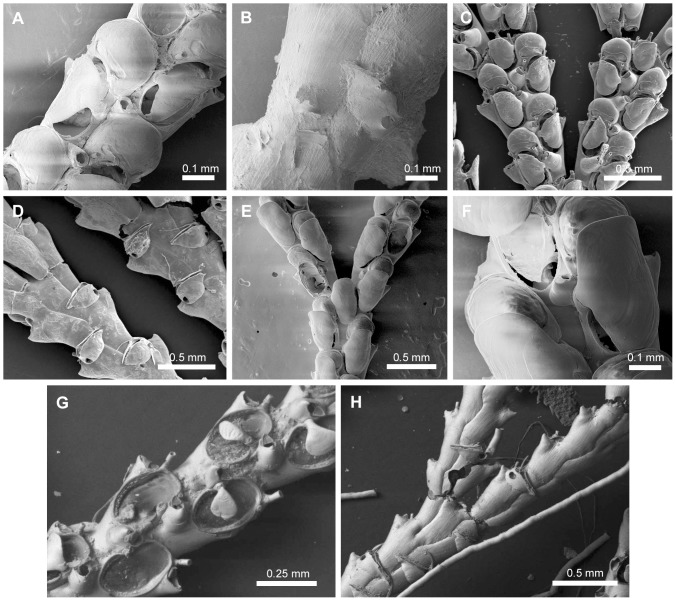
Species assigned to *Aquiloniella* n. gen. Species assigned to *Aquiloniella* n. gen. A–B, *Aquiloniella americana* (Packard, 1863) n. comb., MCZ 134, syntype, ?Labrador. C–D, *Aquiloniella orientalis* (Kluge, 1955) n. comb., Svalbard (P. Kuklinski coll.). E–F, *Aquiloniella paenulata* (Norman, 1903) n. comb., MCZ 523, Gulf of Maine. G–H, *Aquiloniella scabra* (van Beneden, 1848) n. comb., NHMUK 1911.10.1.376, United Kingdom.

urn:lsid:zoobank.org:act:EF783DC7-CA4B-4399-8B91-56F211F913B1

(Figures 11A–H; Text S4)

#### Type species

*Scrupocellaria americana* Packard, 1863 (Figures 11A–B).

#### Diagnosis

Candidae with jointed branches and almost rectangular zooids, with oval opesia occupying about half of the length of the zooid. Joints crossing the gymnocyst of outer and inner zooids at the bifurcation. Cryptocyst reduced around opesia. Oral spines often present, unbranched. Frontal scuta symmetrical, oval, arising at the median region of the inner part of the opesia. Lateral avicularia often present, monomorphic. Frontal avicularia often present, small, monomorphic. Abfrontal avicularia often present, small, with a rhizoidal foramen; setal groove straight, directed transversely; the setal groove is placed distally to the rhizoidal pore. Ooecium with single ectooecial fenestra.

#### Etymology

The genus name refers to the Roman god of the north wind, *Aquilon*, bringer of cold winter air, in allusion to its occurrence in Arctic and sub-Arctic waters, with the Latin diminutive suffix –*iella* (feminine), little, in allusion to its short distal spines.

#### Remarks

*Aquiloniella* n. gen. is erected to include five species reported in Arctic and sub-Arctic waters: *Aq. americana* (Packard, 1863) n. comb. [62] (Figures 11A–B), *Aq. aviculareae* (Yanagi & Okada, 1918) n. comb. [63] (specimens have not been examined using SEM; thus, this species was not included in the phylogeny), *Aq. orientalis* (Kluge, 1955) n. comb. [64] (Figures 11C–D), *Aq. paenulata* (Norman, 1903) n. comb. [65] (Figures 11E–F), and *Aq. scabra* (van Beneden, 1848) n. comb. [66] (Figures 11G–H). *Scrupocellaria scabra* var. *paenulata* forma *minor* Kluge, 1915 has abfrontal avicularia, like those of *Aquiloniella* species, but it is considered a *nomen nudum* [67].

*Aquiloniella* n. gen. is distinguished from *Tricellaria* by absence of the lateral rhizoidal chamber associated to the outer zooids of the bifurcation, presence of abfrontal avicularia and a scutum arising at the median region of the inner part of the opesia. *Aquiloniella* n. gen. is easily set apart from *Scrupocellaria s. str.* in having abfrontal avicularia (rather than abfrontal vibracula in *Scrupocellaria s. str.*) and in the shape of lateral avicularia.

#### Genus Aspiscellaria n. gen

**Figure 12:**
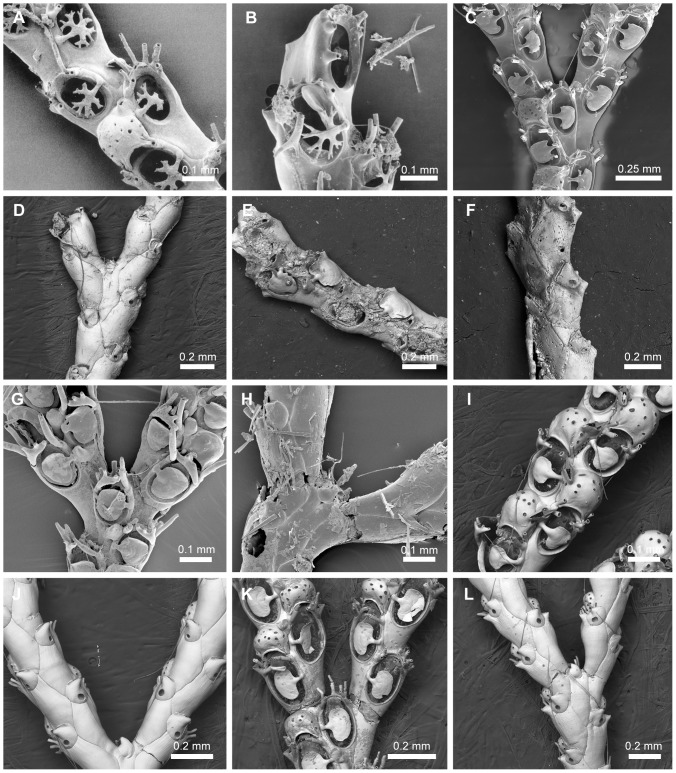
Species assigned to *Aspiscellaria* n. gen. Species assigned to *Aspiscellaria* n. gen. A–B, *Aspiscellaria bellula* (Osburn 1947) n. comb., AMNH 1518.1, Jamaica. C–D, *Aspiscellaria carmabi* (Fransen, 1986) n. comb.; C, VMNH 13699, Belize; D, NHMUK 2012.7.1.10, paratype, Curaçao. E–F, *Aspiscellaria cornigera* (Pourtalès, 1867) n. comb., NHMUK 1911.10.1.368, Barbados. G–H, *Aspiscellaria frondis* (Kirkpatrick 1890) n. comb., LM Vieira coll., Brazil. I–J, *Aspiscellaria hildae* (Fransen, 1986) n. comb., NHMUK 2012.7.1.9, paratype, Curaçao. K–L, *Aspiscellaria piscaderaensis* (Fransen, 1986) n. comb., NHMUK 2012.7.1.8, paratype, Curaçao.

urn:lsid:zoobank.org:act:648B9E0D-6793-4190-85A4-EFA4B4652E50

(Figures 12A–L; Text S5)

#### Type species

*Scrupocellaria piscaderaensis* Fransen, 1986 (Figures 12K–L).

#### Diagnosis

Candidae with jointed branches and almost rectangular zooids, with short oval opesia occupying half-length of the zooid. Joints crossing the gymnocyst of outer and inner zooids at the bifurcation, or at proximal end of the opesia of outer zooids. Cryptocyst variably developed around opesia. Oral spines often present, with proximal-most outer spines branched two or more times (cervicorn). Frontal scuta symmetrical, oval, arising at or slightly below the median region of the inner part of the opesia. Lateral avicularia often present, monomorphic, with a slightly serrated rostrum and straight tip. Frontal avicularia often present, small, monomorphic. Vibracular chamber trapezoidal, with a rhizoidal foramen; setal groove straight, directed transversely; the setal groove is placed distally to the rhizoidal pore; 1 axillary vibraculum with lateral setal groove present. Ooecium with some ectooecial pores.

#### Etymology

The genus names is formed from the word *aspis* (Greek), the generic term for shield, in allusion to its rounded scutum, + *cellaria*, used for some bryozoan genera.

#### Remarks

The vibracular chamber with transverse setal groove resembles those of *Cradoscrupocellaria* and *Pomocellaria* n. gen. *Aspiscellaria* n. gen. is easily distinguished from these two genera by its proximal-most spine which is cervicorn in shape. *Pomocellaria* n. gen. is distinct in the position of the scutum, arising from the distal third of the inner edge of the opesia, and in having an ooecium with a single frontal fenestra.

We reassigned eight species to *Aspiscellaria*: *Ap. bellula* (Osburn, 1947) n. comb. [40] (Figures 12A–B), *Ap. carmabi* (Fransen, 1986) n. comb. [68] (Figures 12C–D), *Ap. cornigera* (Pourtalès, 1867) n. comb. [69] (Figures 12E–F), *Ap. frondis* (Kirkpatrick, 1890) n. comb. [70] (Figures 12G–H), *Ap. hildae* (Fransen, 1986) n. comb. [68] (Figures 12I–J), *Ap. panamensis* (Osburn, 1950) n. comb. [12] (specimens have not been examined using SEM; thus, this species was not included in the phylogeny), *Ap. piscaderaensis* (Fransen, 1986) n. comb. [68] (Figures 12K–L), and *Ap. unicornis* (Liu, 1980) n. comb. [71] (specimens have not been examined using SEM; thus, this species was not included in the phylogeny; despite the lack of comparative specimens available for study, illustrations show this Chinese species also has proximal-most cervicorn spine, porous ooecium and vibraculum with transverse setal groove; see [71]).

Three species of *Aspiscellaria* n. gen. are characterized by the absence of a lateral avicularium: *Ap. frondis*, *Ap. hildae,* and *Ap. unicornis*; these three species are distinguished from each other by the shape of the scutum, the shape and size of the frontal avicularium and the shape of ectooecial pores. *Aspicellaria frondis* has a rounded scutum covering the majority of the opesia, with a narrow cryptocyst, distinct from *Ap. hildae* and *Ap. unicornis*; the two later species are distinct in the size of the zooids and in having shorter frontal avicularia than *Ap. unicornis*. *Aspiscellaria carmabi* is distinguishable from *Ap. piscaderaensis* by the position of joints at the outer zooids at the bifurcation, and shape of frontal avicularium. *Aspiscellaria cornigera* is characterized by a well-developed scutum with projections at its proximal and distal inner edge. The examination of museum specimens of *Aspiscellaria* also revealed that additional new species await description.

#### Genus *Paralicornia* n. gen

**Figure 13:**
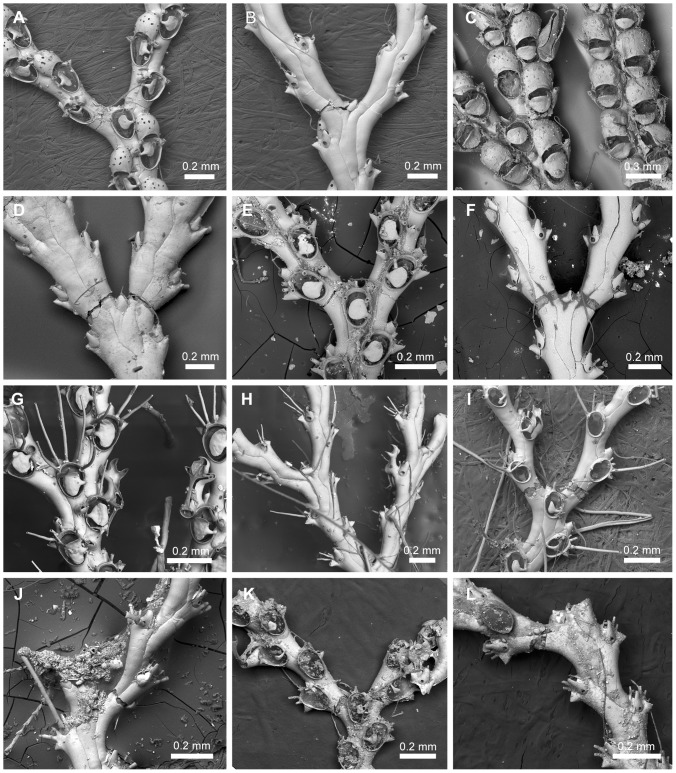
Species assigned to *Paralicornia* n. gen. Species assigned to *Paralicornia* n. gen. A–B, *Paralicornia limatula* (Hayward, 1988) n. comb., NHMUK 1987.1.18.41, Mauritius. C–D, *Paralicornia obtecta* (Haswell, 1880) n. comb., NHMUK 1897.5.1.209, Australia. E–F, *Paralicornia sinuosa* (Canu & Bassler, 1927) n. comb., USNM 8426, Hawaii. G–L, Undescribed species of *Paralicornia*: (G–H) NHMUK 1996.4.26.8, Mauritius; (I–J) NHMUK 1936.12.30.177, Mauritius; (K–L) NHMUK 2000.4.11.1546, Australia.

urn:lsid:zoobank.org:act:A6204B1E-D9BE-4543-9473-E5A33FBD5C29

(Figures 13A–L; Text S6)

#### Type species

*Scrupocellaria sinuosa* Canu & Bassler, 1927 (Figures 13E–F).

#### Diagnosis

Candidae with jointed branches and almost rectangular zooids with short oval opesia occupying half the zooid length. Joints crossing the gymnocyst of outer and inner zooids at the bifurcation. Cryptocyst reduced around opesia. Oral spines present, unbranched. Frontal scuta symmetrical to asymmetrical, oval to subrectangular, arising at the median region (or slightly below) the inner part of the opesia. Lateral avicularia present, with a slightly serrated rostrum and straight tip; lateral avicularium sometimes replaced by an avicularium. Frontal avicularia often present, small, monomorphic. Vibracular chamber almost triangular, with a rhizoidal foramen; setal groove straight, obliquely directed and occupying half of the length of the vibracular chamber; the setal groove is placed distally to the rhizoidal pore; 1 axillary vibraculum with lateral setal groove. Ooecium with some ectooecial pores.

#### Etymology

The generic name refers to the similarities of this genus with some *Licornia* species.

#### Remarks

Vieira *et al.* [23], [24] use two major character differences, *viz.* presence of ooecia with ectooecial pores and single axillary vibraculum, to reassign some species of *Scrupocellaria* to two separate genera, *Licornia* and *Cradoscrupocellaria*. The genus *Cradoscrupocellaria* is quite distinct from *Licornia* and *Paralicornia* n. gen. in having a vibracular chamber with a transversal setal groove; *Cradoscrupocellaria* seems to be morphologically related to *Aspiscellaria*, but the two are distinct in the shape of the frontal scutum (branched in *Cradoscrupocellaria* and rounded in *Aspiscellaria*, excepted by *Ap. belulla*), the presence of dimorphic frontal avicularia (characteristic of some *Cradoscrupocellaria*) and the presence of branched distal spines (characteristic of *Aspiscellaria*). The genus *Licornia* resembles *Paralicornia* n. gen. in the direction of the setal groove of the vibraculum, but the two genera are distinct in the position of the joints in the outer zooids at the bifurcation (crossing the opesia in *Licornia* and the gymnocyst in *Paralicornia* n. gen.), the presence of shorter opesia in *Paralicornia* n. gen. than in *Licornia*, the presence of bifurcated distal spines in *Licornia*, and the length of setal groove, longer in *Licornia* species than those of *Paralicornia*. The gigantic trifoliate lateral avicularia found in the three known species of *Paralicornia* n. gen. seem to be absent in *Licornia*.

Seven species previously assigned to *Scrupocellaria* are transferred here to *Paralicornia* n. gen.: *viz*. *Pa. hamata* (Tilbrook & Vieira, 2012) n. comb. [11] (figured by [11]), *Pa. limatula* (Hayward, 1988) n. comb. [55] (Figures 13A–B), *Pa. obtecta* (Haswell, 1880) n. comb. [72] (Figures 13C–D), *Pa. pusilla* (Smitt, 1872) n. comb. [73] (figured by [74]), *Pa. sinuosa* (Canu & Bassler, 1927) n. comb. [75] (Figures 13E–F; see also [11]), *Pa. spatulatoidea* (Liu, 1980) n. comb. [71] (specimens have not been examined using SEM; thus, this species was not included in the phylogeny), and *Pa. unguiculata* (Osburn, 1950) n. comb. [12] (specimens have not been examined using SEM; thus, this species was not included in the phylogeny). *Paralicornia unguiculata* has distinct dimorphic lateral avicularia with ligulate mandibles.

The examination of specimens deposited at the NHMUK, AMNH and USNM revealed that about a dozen new species of *Paralicornia* will also need to be described (see Figures 13G–L). *Scrupocellaria spatulata* (d'Orbigny, 1851) [2] has been treated as morphologically related to *Pa. sinuosa* [11], but due to the absence of type material and the presence of similar species, this taxon is here considered a *species inquirenda*, *i.e.* a species with doubtful identity needing further assessment.

#### Genus *Pomocellaria* n. gen

**Figure 14:**
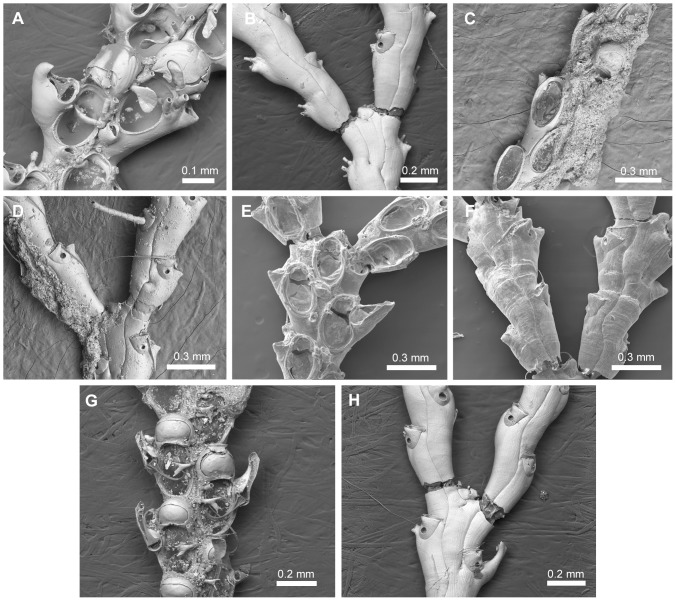
Species assigned to *Pomocellaria* n. gen. Species assigned to *Pomocellaria* n. gen. A–B, *Pomocellaria californica* (Trask, 1857) n. comb., NHMUK 1968.1.18.110, California. C–D, *Pomocellaria inarmata* (O'Donoghue & O'Donoghue, 1926) n. comb., NHMUK 1964.4.2.10, holotype, Pacific coast of United States. E–F, *Pomocellaria talonis* (Osburn, 1950) n. comb., VMNH 13690, Pacific coast of United States. G–H, *Pomocellaria varians* (Hincks, 1882) n. comb., NHMUK 1968.1.18.111, California.

urn:lsid:zoobank.org:act:1E033805-798F-4680-B3F6-BDF303C967E8

(Figures 14A–H; Text S7)

#### Type species

*Scrupocellaria californica* Trask, 1857 (Figures 14A–B).

#### Diagnosis

Candidae with jointed branches and almost rectangular zooids, with oval opesia occupying half-length of the zooid. Joints crossing the gymnocyst of outer and inner zooids at the bifurcation. Cryptocyst reduced around opesia. Oral spines often present, unbranched. Frontal scuta arising at the distal third of the inner part of the opesia. Lateral avicularia often present, dimorphic. Frontal avicularia often present, small, monomorphic. Vibracular chamber trapezoidal, with a rhizoidal foramen; setal groove straight, directed transversely, placed distally to the rhizoidal pore; an axillary vibraculum with lateral setal groove. Ooecium with single ectooecial fenestra.

#### Etymology

The generic name is composed from *pomo* (an indigenous people of California) + *cellaria*, used for some bryozoan genera.

#### Remarks

*Pomocellaria* n. gen. is erected to include four Eastern Pacific species: *Po. californica* (Trask, 1857) n. comb. [76] (Figures 14A–B;  = *Scrupocellaria brevisetis* Hincks, 1882 [77]; see [78]), *Po. inarmata* (O'Donoghue & O'Donoghue, 1926) n. comb. [79] (Figures 14C–D), *Po. talonis* (Osburn, 1950) n. comb. [12] (Figures 14E–F), and *Po. varians* (Hincks, 1882) n. comb. [77] (Figures 14G–H). *Pomocellaria* n. gen. is distinguishable from the other two genera with a trapezoidal vibracular chamber—*i.e. Cradoscrupocellaria* and *Aspiscellaria* n. gen.—in the position of the scutum, arising from the distal third of the inner edge of the opesia rather than the median part of the inner edge of the opesia; and in having an ooecium with a single frontal fenestra rather than one with many pseudopores. The dimorphic lateral avicularium and distal spines are absent in *Pomocellaria inarmata*.

#### Genus *Scrupocaberea* n. gen

**Figure 15:**
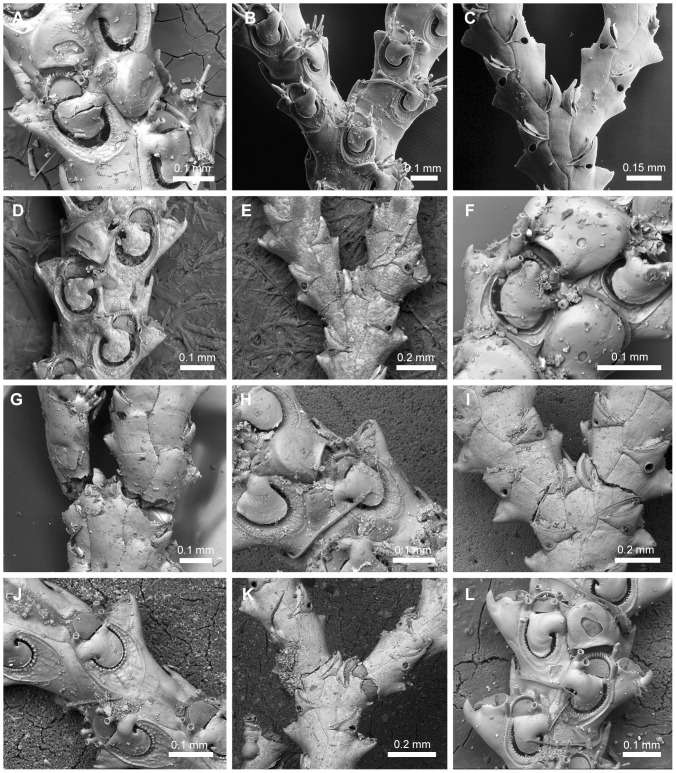
Species assigned to *Scrupocaberea* n. gen. Species assigned to *Scrupocaberea* n. gen. A–C, *Scrupocaberea maderensis* (Busk, 1860) n. comb. A, NHMUK 1899.7.1.780, syntype, Madeira; B–C, specimen from Azores. D–E, *Scrupocaberea dongolensis* (Waters, 1909) n. comb., NHMUK 1928.9.13.98, syntype, Sri Lanka. F–G, *Scrupocaberea ornithorhynchus* (Wyville Thomson, 1858) n. comb., NHMUK 1899.7.1.783, syntype, Australia. H–L, Undescribed species of *Scrupocaberea*: (H–I) NHMUK 1887.12.9.103, Cape Verde, (J–K) NHMUK 1961.11.2.42, Gulf of Mexico; (L) NHMUK 1928.3.6.169, Malay Archipelago.

urn:lsid:zoobank.org:act:03E75D59-E2D3-45E5-83F0-412CFC7ABCE2

(Figures 15A–L; Text S8)

#### Type species

*Scrupocellaria maderensis* Busk, 1860 (Figures 15A–C).

#### Diagnosis

Candidae with jointed branches, almost rectangular zooids, broadly oval opesia occupying most of the frontal surface, and a truncate distal opercular area. Operculum well-chitinized. Joints crossing the gymnocyst of outer and inner zooids at the bifurcation. Cryptocyst present, well developed around the opesia. Oral spines present, unbranched. Frontal scuta asymmetrical, with a stout base, more developed proximally than distally, arising from the distal third of the inner margin of the opesia, below the most proximal inner spine. Lateral avicularia present, aquiline, with a serrated rostrum and hooked tip. Frontal avicularia often present, small, monomorphic. Vibracular chamber almost triangular, with a rhizoidal foramen; setal groove curved and directed obliquely; 2 axillary vibracula. Ooecium with single and large ectooecial fenestra and a small avicularium at its outer border. Distal edge of ovicelled zooid with toothed rim.

#### Etymology

The generic name refers to the mixture of morphologic characteristics of the new genus with *Scrupocellaria* and *Caberea* species.

#### Remarks

The presence of a well-chitinized operculum placed in an obliquely truncate distal area, a toothed rim on the distal edge of ovicelled zooids, and the presence of two axillary vibracula led us to include four species in a new genus, *Scrupocaberea* n. gen.: *Sb. dongolensis* (Waters, 1909) n. comb. [80] (Figures 15D–E), *Sb. gilbertensis* (Maplestone, 1909) n. comb. [81] (the type specimens could not be figured), *Sb. maderensis* (Busk, 1860) n. comb. [82] (Figures 15A–C), and *Sb. ornithorhynchus* (Thomson, 1858) n. comb. [83] (Figures 15F–G).

*Scrupocaberea maderensis* has been reported to be widespread in tropical and subtropical waters worldwide [6], [26], but re-examination of some of the NHMUK specimens so identified, revealed that this name represents a species complex (*e.g.* Figures 15H–L). At least two species were previously synonymized under *Sb. maderensis* by Harmer [26], *viz. Scrupocaberea dongolensis* and *Sb. gilbertensis*; these species are distinct from *Sb. maderensis* (Figures 15A–C) by virtue of the number of oral spines, shape of the scutum (smaller in *Sb. dongolensis* and larger in *Sb. gilbertensis* than *Sb. maderensis*), shape of frontal avicularia, surface of the cryptocyst (granulose in *Sb. gilbertensis*), and size of the autozooids (smaller in *Sb. dongolensis* and *Sb. gilbertensis* than those of *Sb. maderensis*). Zooids of *Sb. ornithorhynchus* also have a well-chitinized operculum; this species differs from *Sb. maderensis* in having a vibracular chamber with a shorter setal groove and by the shape of scutum which is asymetrically developed in distal edge in *Sb. ornithorhynchus* rather than truncate as those of *Sb. maderensis*.

### Remarks on other species previously assigned to *Scrupocellaria*

Four Recent species of *Scrupocellaria* not included in this study probably belong to *Licornia* due to their having porous ooecia and similarly shaped abfrontal vibracula; they are here reassigned to *Licornia*: *Licornia mexicana* (Osburn, 1950) n. comb. [12], *Licornia pugnax* (Osburn, 1950) n. comb. [12], *Licornia spinigera* (Osburn, 1950) n. comb. [12], and *Licornia wasinensis* (Waters, 1913) n. comb. [39]. In *L. spinigera* the joints pass across proximal end of the opesia of outer zooids at the bifurcation, but pass more distally (near to the half-length of opesia) in *L. mexicana* and *L. pugnax*. *Licornia wasinensis* is characterized by the absence of a scutum and by dimorphic lateral avicularia with forked mandibles; this forked mandible [39] is distinguishable from the trifoliate mandible characteristic of *Paralicornia* species.

We have examined the type specimens of *Scrupocellaria elegantissima* David & Pouyet, 1986 [84] (MNHN 13098, holotype; MNHN 13131–3, paratypes); this species is here reassigned to *Notoplites* Harmer, 1923 due to the presence of basal avicularia and joints passing across zooids FJ and GK at bifurcation [25], thus *Notoplites elegantissima* (David & Pouyet, 1986) n. comb.

Three Recent species (specimens have not been examined using SEM; thus, these species were not included in the phylogeny) are unassigned to any genus of Candidae, *Scrupocellaria micheli* Marcus, 1955 [85] (no specimens have been found), *Scrupocellaria profundis* Osburn, 1950 [12] (SBMNH 96161, balsam slide, paratype; specimens have not been examined using SEM), and *Scrupocellaria uniseriata* Liu, 1984 [86] (no specimens have been found). The Brazilian species *Scrupocellaria micheli* is distinct in the irregular branching pattern of the colony and the presence of large aquiline lateral avicularia [85]. We suggest a morphological relationship between *Scrupocellaria micheli* and other *Paralicornia* n. gen. and *Licornia* species due to the presence of vibracula with a straight, obliquely directed setal groove occupying half of the length of the vibracular chamber, and an ooecium with some ectooecial pores; this species is tentatively assigned to *Licornia*, thus *Licornia micheli* (Marcus, 1955) n. comb. *Scrupocellaria profundis* Osburn, 1950 is a deep water species (recorded from more than 1000 m deep [12]), characterized by the presence of two axial vibracula and no scutum, as *Sl. scruposa*; this species is distinguishable from any other members of genus (as well as other genera described above) in the shape of its zooids, which are twisted at the axis of the maternal internode, and the position of the radicles chamber, lateral rather than proximally placed in the vibracular chamber and two axial vibracula with longitudinal straight setal groove (setal groove are curved in other genera with two axial vibracula, *viz. Canda*, *Scrupocellaria* and *Scrupocaberea* n. gen.). *Bathycellaria* n. gen. (urn:lsid:zoobank.org:act:362C7211-AE4D-4430-A385-B768B2E06FE8) (from the Greek word *bathys*, deep, in allusion of its occurrence in deep sea, + *cellaria*, used for some bryozoan genera; Gender, feminine) is erected to accommodate Osburn's species [12] (type species by monotypy), thus *Bathycellaria profundis* (Osburn, 1950) n. comb. *Scrupocellaria uniseriata* Liu, 1984 has unique uniserial colonies [86], distinct from other genera of Candidae; *Sinocellaria* n. gen. (urn:lsid:zoobank.org:act:93013294-FD7C-40D4-8361-370CF74B631D) (from *sino*-, meaning from China, + *cellaria*, used for some bryozoan genera; Gender, feminine) is erected to accommodate Liu's (1984) species (type species by monotypy), thus *Sinocellaria uniseriata* (Liu, 1984) n. comb.

About 19 fossil species have been assigned to the genus *Scrupocellaria*. *Scrupocellaria clausa* Canu & Bassler, 1920 [87] (USNM 64247–8, Oligocene Vicksburgian, syntypes) belongs to *Notoplites*, but this name is preoccupied by *Notoplites clausus* (Busk, 1884) [88]; thus, *Notoplites americanus* n. name (urn:lsid:zoobank.org:act:C032937B-14AF-415D-86B4-E4270574EDFE) is proposed as a replacement name for *Scrupocellaria clausa* Canu & Bassler, 1920. Three species described from the Oligocene (Vicksburgian) Alabama, USA, are reassigned to *Canda*: *Canda rathbuni* (Canu & Bassler, 1920) n. comb. [87] (USNM 64245, holotype), *Canda triangulata* (Canu & Bassler, 1920) n. comb. [87] (USNM 64241, holotype) and *Canda williardi* (Canu & Bassler, 1920) n. comb. [87] (USNM 64243, syntypes). At least four species, *Scrupocellaria cookei* Canu & Bassler, 1920 [87] (USNM 64237, Oligocene Vicksburgian, syntypes), *Scrupocellaria milneri* Canu & Bassler, 1920 [87] (USNM 64238–40, Oligocene Vicksburgian, syntypes), *Scrupocellaria raigadensis* Badve & Sonar, 1997 (Holocene, India; see [89] for the descriptions and figures), *Scrupocellaria resseri* Canu & Bassler, 1920 [87] (USNM 64242, Oligocene Vicksburgian, syntypes), belong to *Licornia*, thus *Licornia cookie* (Canu & Bassler, 1920) n. comb., *Licornia milneri* (Canu & Bassler, 1920) n. comb., *Licornia raigadensis* (Badve & Sonar, 1997) n. comb., and *Licornia resseri* (Canu & Bassler, 1920) n. comb. *Scrupocellaria marosticana* Bizzarini & Braga, 2001 [90] (new name for *Scrupocellaria watersi* Bizzarini & Braga, 1999 [91] non *Scrupocellaria watersi* Kluge, 1914 [92]) is distinguishable from other fossils in the group in having avicularia on the abfrontal surface of the colony; it is reassigned to *Aquiloniella*, thus *Aquiloniella marosticana* (Bizzarini & Braga, 2001) n. comb. The characteristics observed in the type specimens of *Scrupocellaria dubia* Canu & Bassler, 1920 [87] (USNM 63953, Eocene Jacksonian, holotype) and *Scrupocellaria vaughani* Canu & Bassler, 1920 [87] (USNM 64244, Oligocene Vicksburgian, holotype), did not allow us to assign the species to any genus of the Candidae. The assignment of eight species—*viz. Scrupocellaria elliptica* Reuss, 1869 [93], *Scrupocellaria brendolensis* Waters, 1891 [94], *Scrupocellaria crenulata* MacGillivray, 1895 [95], *Scrupocellaria elliptica* (Reuss, 1848) [96], *Scrupocellaria gracilis* Reuss, 1869 [93], *Scrupocellaria montecchiensis* Waters, 1891 [94], *Scrupocellaria prolifera* (d'Orbigny, 1853) [2] and *Scrupocellaria rostrata* Malecki, 1980 [97]—are still uncertain and examination of the type specimens of these species will be required to confirm their assignment in *Scrupocellaria s. str*. or other genera.

## Supporting Information

Table S1 Character matrix used in phylogenetic analysis.
(XLSX)

Text S1 List of specimens examined and included in the phylogenetic analysis.
(DOCX)

Text S2 List of character state optimisations for semi-strict consensus tree (Figure 1). No List of autapomorphies was provided.
(DOCX)

Text S3 List of type material of *Scrupocellaria* species.
(DOCX)

Text S4 List of type material of *Aquiloniella* species.
(DOCX)

Text S5 List of type material of *Aspiscellaria* n. gen.
(DOCX)

Text S6 List of type material of *Paralicornia* n. gen.
(DOCX)

Text S7 List of type material of *Pomocellaria* n. gen.
(DOCX)

Text S8 List of type material of *Scrupocaberea* n. gen.
(DOCX)
