# Supplementary material for: Evidence for Polyphyly of the Genus Scrupocellaria (Bryozoa: Candidae) Based on a Phylogenetic Analysis of Morphological Characters
Source: PLoS One. 2014 Apr 18;9(4):e95296. doi: 10.1371/journal.pone.0095296 (PMC3991637; doi:10.1371/journal.pone.0095296)
Supplement: Text S1 — List of specimens examined and included in the phylogenetic analysis. (DOCX) [file pone.0095296.s002.docx]

**Evidence for polyphyly of the genus *Scrupocellaria* (Bryozoa: Candidae) based on a phylogenetic analysis of morphological characters**

**Leandro M. Vieira^1^*, Mary E. Spencer Jones^2^, Judith E. Winston^3^, Alvaro E. Migotto^1^, Antonio C. Marques^4^**

**1** Centro de Biologia Marinha, Universidade de São Paulo, São Sebastião, SP, Brazil, **2** Department of Life Sciences, Natural History Museum, London, UK, **3** Virginia Museum of Natural History, Martinsville, VA, USA, **4** Departamento de Zoologia, Instituto de Biociências, Universidade de São Paulo, SP, Brazil

*Correspondent author. Email: leandromanzoni@hotmail.com

**Supporting Information Text S1 - List of specimens examined and included in the phylogenetic analysis.**

*Aquiloniella* *americana* (Packard, 1863). MCZ 134, ?Labrador, syntype.

*Aquiloniella* *orientalis* (Kluge, 1955). P. Kuklinski coll. (pers. comm.), Spitsbergen, Svalbard, non-type.

*Aquiloniella* *paenulata* (Norman, 1903). MCZ 523, Labrador, Canada, non-type.

*Aquiloniella* *scabra* (van Beneden, 1848). NHMUK 1911.10.1.376, British Isles, non-type.

*Aspiscellaria* *carmabi* (Fransen, 1986). RMNH 02977, Curaçao, holotype. RMNH 03042–52, Curaçao, paratypes.

*Aspiscellaria* *cornigera* (Pourtalès, 1867). NHMUK 1911.10.1.368, Barbados, non-type.

*Aspiscellaria* *frondis* (Kirkpatrick, 1890). NHMUK 1888.4.16.20, Fernando de Noronha, Brazil, holotype. NHMUK 1931.12.19.7, Dry Tortugas, USA; LMV collection, Alagoas, Brazil, non-types.

*Aspiscellaria* *hamata* (Tilbrook & Vieira, 2012). MTQ G25155, Queensland, Australia, holotype.

*Aspiscellaria* *hildae* (Fransen, 1986). RMNH 02980, Curaçao, holotype. RMNH 03065–70, Curaçao, paratypes.

*Aspiscellaria* *piscaderaensis* (Fransen, 1986). RMNH 02979, Curaçao, holotype. RMNH 03053–62, Curaçao, paratypes.

*Caberea boryi* (Audouin, 1826). NHMUK 1881.4.29.11, Italy; NHMUK 1937.9.28.6, Red Sea, non-types.

*Caberea* sp. LMV coll., Rio de Janeiro, Brazil, non-type.

*Canda retiformis* Pourtalès, 1867. MCZ 167, Florida, USA, syntypes.

*Canda clypeata* (Haswell, 1880). NHMUK 1910.6.16.1, Australia, paratype.

*Cradoscrupocellaria aegyptiana* Vieira, Spencer Jones & Winston, 2013. NHMUK 1963.8.2.16, Egypt, holotype.

*Cradoscrupocellaria arisaigensis* Vieira, Spencer Jones & Winston, 2013. NHMUK 2010.12.6.1, Arisaig, Scotland, holotype.

*Cradoscrupocellaria atlantica* Vieira, Spencer Jones & Winston, 2013. MZUSP 532, São Paulo, Brazil, holotype. MZUSP 533–540, São Paulo, Brazil, paratypes. VMNH 10403.0000, Florida, USA, non-type.

*Cradoscrupocellaria bertholletii* (Audouin, 1826). NHMUK 1926.9.6.58, Suez Canal, neotype. NHMUK 1899.7.1.736, Mediterranean, non-type.

*Cradoscrupocellaria calypso* Vieira, Spencer Jones & Winston, 2013. MNHN 15979, Pernambuco, Brazil, holotype. NHMUK 2013.4.10.1, Pernambuco, Brazil, paratype.

*Cradoscrupocellaria curacaoensis* (Fransen, 1986). RMNH 02975, Curaçao, holotype. NHMUK 2012.7.1.12, Curação; RMNH 03034–03041, Curação; RMNH 03064, Curação, paratypes.

*Cradoscrupocellaria ellisii* (Vieira & Spencer Jones, 2012). NHMUK 1911.10.1.353, British Isles, holotype.

*Cradoscrupocellaria floridana* Vieira, Spencer Jones & Winston, 2013. NHMUK 2010.12.6.2, Florida, USA, holotype. NHMUK 1931.12.19.3–4, Florida, USA; NHMUK 1935.11.26.1, Florida, USA, paratypes.

*Cradoscrupocellaria galapagensis* Vieira, Spencer Jones & Winston, 2013. NHMUK 2010.12.6.3, Galapagos, holotype. NHMUK 1924.4.26.243, Galapagos; NHMUK 1924.4.26.286, Galapagos, paratypes.

*Cradoscrupocellaria gautieri* Vieira, Spencer Jones & Winston, 2013. NHMUK 1882.5.24.9, Algeria, holotype. NHMUK 1882.5.24.8–12, Algeria, paratypes.

*Cradoscrupocellaria gorgonensis* Vieira, Spencer Jones & Winston, 2013. NHMUK 2010.12.6.4, Gorgona, Colombia, holotype. NHMUK 1929.4.26.19, Gorgona, Colombia; NHMUK 2010.12.6.5–7, Gorgona, Colombia, paratypes.

*Cradoscrupocellaria hastingsae* Vieira, Spencer Jones & Winston, 2013. NHMUK 2010.12.6.8, Galapagos, holotype. NHMUK 1929.4.26.44, Galapagos; NHMUK 1929.4.26.25, Galapagos; NHMUK 2010.12.6.9–12, Galapagos, paratypes.

*Cradoscrupocellaria hirsuta* (Jullien & Calvet, 1903). MOM 420323, Azores, lectotype. NHMUK 1911.10.1.386, Madeira; NHMUK 2012.7.1.1, Azores, non-type.

*Cradoscrupocellaria insularis* Vieira, Spencer Jones & Winston, 2013. NHMUK 2010.12.6.16, Cape Verde, holotype. NHMUK 1899.7.1.837, Cape Verde; NHMUK 2010.10.1.7–8, Cape Verde, paratypes.

*Cradoscrupocellaria jamaicensis* Vieira, Spencer Jones & Winston, 2013. AMNH 1522.1, Jamaica, holotype. AMNH 1524.1, Jamaica; AMNH 1529.1, Jamaica, paratypes.

*Cradoscrupocellaria lagaaiji* Vieira, Spencer Jones & Winston, 2013. NHMUK 1975.7.18.31, France (Mediterranean), holotype.

*Cradoscrupocellaria macrorhyncha* (Gautier, 1962). NHMUK 1965.9.2.4, Mediterranean, lectotype. NHMUK 1874.4.25.34 Italy; NHMUK 1975.1.12.434, Aegean Sea, non-types.

*Cradoscrupocellaria macrorhynchoides* Vieira, Spencer Jones & Winston, 2013. NHMUK 2010.12.6.19, Queensland, Australia, holotype. NHMUK 2010.12.6.20, Queensland, Australia, paratype.

*Cradoscrupocellaria makua* Vieira, Spencer Jones & Winston, 2013. NHMUK 2010.12.6.17, Mozambique, holotype. NHMUK 1938.5.2.4, Mozambique; NHMUK 2010.12.6.18, Mozambique, paratypes.

*Cradoscrupocellaria marcusorum* Vieira, Spencer Jones & Winston, 2013. NHMUK 2010.12.6.28, São Paulo, Brazil, holotype. NHMUK 1948.2.16.46, São Paulo, Brazil, paratype.

*Cradoscrupocellaria normani* Vieira, Spencer Jones & Winston, 2013. NHMUK 1911.10.1.355, Madeira, holotype.

*Cradoscrupocellaria odonoghuei* Vieira, Spencer Jones & Winston, 2013. NHMUK 2010.12.6.21, Scotland, holotype. NHMUK 2010.12.6.22, Scotland, paratype.

*Cradoscrupocellaria osburni* Vieira, Spencer Jones & Winston, 2013. NHMUK 2010.6.14.3, Panama, holotype.

*Cradoscrupocellaria reptans* (Linnaeus, 1758). LSL 1248.31.I, lectotype. NHMUK 1963.3.6.35, British Isles, non-type.

*Cradoscrupocellaria tenuirostris* (Osburn, 1950). SBMNH 96158, Gulf of California, paratype. NHMUK 2010.10.5.1, California, USA, non-type.

*Licornia annectens* (MacGillivray, 1887). NMV F.45606.1–3, Indonesia, syntypes.

*Licornia cervicornis* (Busk, 1852a). NHMUK 1854.11.15.81, Queensland, Australia, holotype. NHMUK 1899.7.1.4552–3, Australia, non-type.

*Licornia cyclostoma* (Busk, 1852a). NHMUK 1854.11.15.77, Victoria, Australia, holotype. NHMUK 1899.6.1.340, Port Phillips, Australia, non-type.

*Licornia curvata* (Harmer, 1926). RMNH (ZMA 01063aq), holotype; NHMUK 1928.3.6.187, part of holotype.

*Licornia diadema* (Busk, 1852) NHMUK 1854.11.15.50, Queensland, Australia, holotype.

*Licornia diegensis* (Robertson, 1905). NHMUK 2010.12.9.3, California, USA, non-type.

*Licornia drachi* (Marcus, 1955), MZUSP 532, Espírito Santo, Brazil, non-type.

*Licornia ferox* (Busk, 1852). NHMUK 1854.11.15.76, Louisiade Archipelago, holotype. NHMUK 1928.3.6.156, Java, Malay Archipelago, non-type.

*Licornia gaspari* (Thornely, 1907). NHMUK 1936.12.30.126, Gaspar Strait, Indonesia; NHMUK 1936.12.30.136, Gaspar Strait, Indonesia; NHMUK 1936.12.30.146, Gaspar Strait, Indonesia, syntypes.

*Licornia jolloisii* (Audouin, 1826) NHMUK 1926.9.6.84, Red Sea, neotype. NHMUK 1926.9.6.85–94, Red Sea, non-type.

*Licornia longispinosa* (Harmer, 1926). RMNH 00054, Indonesia, schizoholotype; NHMUK 1928.3.6.189–90, Indonesia, schizoholotype; USNM 9389, Indonesia, schizoholotype.

*Licornia macropora* (Osburn, 1950). SBMNH 96151, California, USA; USNM 553519–553520, California, USA, paratypes.

*Licornia peltata* (Tilbrook & Vieira, 2012) MTQ G25998, Queensland, Australia, holotype.

*Licornia prolata* (Tilbrook & Vieira, 2012). MTQ G25341, Queensland, Australia, holotype.

*Licornia regularis* (Osburn, 1940). MCZ 38, Florida, USA; MCZ 40, Florida, USA; MCZ 163, Florida, USA; USNM 2347, Florida, USA, syntypes.

*Licornia securifera* (Busk, 1884). NHMUK 1887.12.9.113–114, Admiralty Island, Papua New Guinea, syntypes.

*Licornia tridentata* (Waters, 1918). MOM 420807, Cape Verde (syntype of *Scrupocellaria cervicornis spinosa* Calvet, 1931).

*Notoplites marsupiatus* (Jullien, 1882). MNHN 2817, Galicia Bank, lectotype.

*Notoplites clausus* (Busk, 1884) NHMUK 1887.12.9.83, Azores, lectotype.

*Paralicornia* *limatula* (Hayward, 1988). NHMUK 1987.1.18.41, Mauritius, holotype.

*Paralicornia* *obtecta* (Haswell, 1880). NHMUK 1928.9.13.103, Queensland, Australia, syntype.

*Paralicornia* *pusilla* (Smitt, 1872). MCZ 0100, Tortugas, USA, syntypes.

*Paralicornia* *sinuosa* (Canu & Bassler, 1927). USNM 8426, Hawaii, holotype; MTQ G25291, Queensland, Australia, non-type.

*Pomocellaria californica* (Trask, 1857). NHMUK 2010.12.8.1, California, USA, non-type.

*Pomocellaria inarmata* (O’Donoghue & O’Donoghue, 1926). NHMUK 1964.4.2.10, NW Pacific, holotype.

*Pomocellaria talonis* (Osburn, 1950). SBMNH 96168, Panama, holotype.

*Pomocellaria varians* (Hincks, 1882). NHMUK 1886.3.6.12, Queen Charlotte, Canada, holotype. NHMUK 1962.6.16.4, California, USA; NHMUK 1968.1.18.111, California, USA; NHMUK 2010.12.8.2, Calornia, USA, non-types.

*Scrupocaberea dongolensis* (Waters, 1909). NHMUK 1928.9.13.98, Sri Lanka, syntype.

*Scrupocaberea gilbertensis* (Maplestone, 1909). MV 45061, Gilbert Island, holotype.

*Scrupocaberea maderensis* (Busk, 1860). NHMUK 1899.7.1.780, Madeira, syntype. NHMUK 1911.10.1.388, Madeira; NHMUK 1922.9.6.1, Madeira, non-types.

*Scrupocaberea ornithorhynchus* (Wyville Thomson, 1858). NHMUK 1899.7.1.783, Bass Strait, Australia, holotype.

*Scrupocellaria aegeensis* Harmelin, 1969. NHMUK 2010.12.7.3, Strait of Scarpanto, Mediterranean; NHMUK 2010.12.7.4, Strait of Scarpanto, Mediterranean, syntypes.

*Scrupocellaria delilii* (Audouin, 1826). NHMUK 2010.12.8.3, Adriatic, non-type.

*Scrupocellaria harmeri* Osburn, 1947. SBMNH 95952, Aruba Island, holotype; SBMNH 95953, Aruba Island, paratype.

*Scrupocellaria incurvata* Waters, 1897. NHMUK 1899.7.1.303, Mediterranean, non-type.

*Scrupocellaria intermedia* Norman, 1893. NHMUK 1911.10.1.369, Trondhjem Fjord, Norway; NHMUK 1912.12.21.835, Trondhjem Fjord, Norway, syntypes.

*Scrupocellaria inermis* Norman, 1867. NHMUK 1911.10.1.367, Scotland; NHMUK 1912.12.21.834, Scotland; NHMUK 1912.12.21.8334, Scotland, syntypes.

*Scrupocellaria minuta* (Kirkpatrick, 1888). NHMUK 1888.1.25.2A, Mauritian, lectotype.

*Scrupocellaria scrupea* Busk, 1851. NHMUK 1854.11.15.79, SW coast of UK, holotype; NHMUK 2010.12.8.5, Guersey, non-type.

*Scrupocellaria scruposa* (Linnaeus, 1758). NHMUK 1888.12.21.57, Scotland; NHMUK 1966.1.10.9, English Channel, non-types.

*Tricellaria arctica* (Busk, 1855). NHMUK 1899.7.1.651–2, Greenland, syntypes.

*Tricellaria elongata* (Smitt, 1868). NHMUK 1911.10.1.379, Greenland; NHMUK 1911.10.1.384, Greenland, non-types.

*Tricellaria gracilis* (van Beneden, 1848) VMNH 11493.0000, VMNH 11570.0000, VMNH 11628.0000, VMNH 11629.0000, VMNH 11646.0000, VMNH 11661.0000, VMNH 12500.0001, VMNH 12501.0000, VMNH 12502.0000, VMNH 12617.0000, N. Atlantic, non-types.

*Tricellaria ternata* (Ellis & Solander, 1786). VMNH 11646.0000, VMNH 11647.0001, VMNH 11662.0003, VMNH 11663.0000, VMNH 11667.0001, VMNH 11669.0002, VMNH 11673.0000, N. Atlantic, non-types.

*?Tricellaria* *congesta* (Norman, 1903). NHMUK 1911.10.1.385, Alaska, holotype.
