# Supplementary material for: Evidence for Polyphyly of the Genus Scrupocellaria (Bryozoa: Candidae) Based on a Phylogenetic Analysis of Morphological Characters
Source: PLoS One. 2014 Apr 18;9(4):e95296. doi: 10.1371/journal.pone.0095296 (PMC3991637; doi:10.1371/journal.pone.0095296)
Supplement: Text S2 — List of character state optimisations for semi-strict consensus tree (Figure 1). No List of autapomorphies was provided. (DOCX) [file pone.0095296.s003.docx]

**Evidence for polyphyly of the genus *Scrupocellaria* (Bryozoa: Candidae) based on a phylogenetic analysis of morphological characters**

**Leandro M. Vieira^1^*, Mary E. Spencer Jones^2^, Judith E. Winston^3^, Alvaro E. Migotto^1^, Antonio C. Marques^4^**

**1** Centro de Biologia Marinha, Universidade de São Paulo, São Sebastião, SP, Brazil, **2** Department of Life Sciences, Natural History Museum, London, UK, **3** Virginia Museum of Natural History, Martinsville, VA, USA, **4** Departamento de Zoologia, Instituto de Biociências, Universidade de São Paulo, SP, Brazil

*Correspondent author. Email: leandromanzoni@hotmail.com

**Supporting Information Text S2 - List of character state optimisations for semi-strict consensus tree (Figure 1). No list of autapomorphies was provided.**

**Node 1.** Character 3: 1→0; Character 4: 1→0; Character 8: 1→0; Character 10: 1→0; Character 17: 4→0; Character 21: 0→1.

**Node 2.** Character 14: 0→1.

**Node 3.** Character 24: 0→1.

**Node 4.** Character 15: 2→1; Character 26: 0→2; Character 27: 1→0.

**Node 5.** Character 21: 1→0.

**Node 6.** Character 15: 1→0.

**Node 7.** Character 15: 0→2; Character 22: 0→1.

**Node 8.** Character 14: 0→1; Character 26: 1→0.

**Node 9.** Character 14: 0→1; Character 24: 1→2.

**Node 10.** Character 12: 1→3.

**Node 11.** Character 16: 4→1; Character 23: 0→1.

**Node 12.** Character 5: 0→1.

**Node 13.** Character 20: 0→1.

**Node 14.** Character 11: 0→1; Character 20: 1→0.

**Node 15.** Character 11: 1→0.

**Node 16.** Have no synapomorphies.

**Node 17.** Character 16: 1→3.

**Node 18.** Character 19: 1→0.

**Node 19.** Character 2: 0→1.

**Node 20.** Character 16: 1→2.

**Node 21.** Character 5: 1→0.

**Node 22.** Character 2: 1→0.

**Node 23.** Character 29: 0→1

**Node 24.** Character 22: 0→2

**Node 25.** Character 5: 0→1; Character 28: 0→1; Character 30: 2→3; Character 32: 0→1.

**Node 26.** Character 2: 0→1; Character 11: 0→1.

**Node 27.** Character 2: 1→0; Character 12: 1→2; Character 30: 3→4; Character 33: 0→1.

**Node 28.** Character 11: 1→0; Character 15: 1→0; Character 23: 0→1.

**Node 29.** Character 1: 0→1.

**Node 30.** Character 2: 1→0; Character 15: 1→2; Character 18: 0→1; Character 21: 0→2; Character 24: 2→0; Character 25: 0→1; Character 28: 1→2; Character 30: 3–1; Character 31: 0→1; Character 35: 1→0.

**Node 31.** Character 5: 1→0; Character 11: 1→0; Character 30: 1→0.

**Node 32.** Character 11: 1→0; Character 30: 1→0.

**Node 33.** Character 14: 1→0.

**Node 34:** Character 2: 0→1.

**Node 35.** Character 8: 0→1; Character 10: 0→1; Character 13: 1→0; Character 14: 1→0; Character 17: 0→2; Character 21: 2→0; Character 30: 1→5; Character 31: 1→0; Character 33: 0→1; Character 34: 0→1; Character 35: 0→1.

**Node 36.** Character 1: 0→1; Character 4: 0→1; Character 6: 0→1; Character 7: 0→1; Character 14: 1→0; Character 15: 2→1; Character 16: 4→5; Character 19: 1→0; Character 33: 0→1.

**Node 37.** Character 5: 1→0; Character 8: 0→1; Character 9: 0→1; Character 10: 0→1; Character 28: 2→1.

**Node 38.** Character 17: 0→2.
