# Supplementary material for: Evidence for Polyphyly of the Genus Scrupocellaria (Bryozoa: Candidae) Based on a Phylogenetic Analysis of Morphological Characters
Source: PLoS One. 2014 Apr 18;9(4):e95296. doi: 10.1371/journal.pone.0095296 (PMC3991637; doi:10.1371/journal.pone.0095296)
Supplement: Text S3 — List of type material of Scrupocellaria species. (DOCX) [file pone.0095296.s004.docx]

**Evidence for polyphyly of the genus *Scrupocellaria* (Bryozoa: Candidae) based on a phylogenetic analysis of morphological characters**

**Leandro M. Vieira^1^*, Mary E. Spencer Jones^2^, Judith E. Winston^3^, Alvaro E. Migotto^1^, Antonio C. Marques^4^**

**1** Centro de Biologia Marinha, Universidade de São Paulo, São Sebastião, SP, Brazil, **2** Department of Life Sciences, Natural History Museum, London, UK, **3** Virginia Museum of Natural History, Martinsville, VA, USA, **4** Departamento de Zoologia, Instituto de Biociências, Universidade de São Paulo, SP, Brazil

*Correspondent author. Email: leandromanzoni@hotmail.com

**Supporting Information Text S3 - List of type material of *Scrupocellaria* species.**

1. *Scrupocellaria aegeensis* Harmelin, 1969

*Scrupocellaria maderensis aegeensis* Harmelin, 1969: 1195, figs. 2.5–6 [47]. *Type locality*: Aegean Sea. *Syntypes*: NHMUK 2010.12.7.3–2, dry, N.O. *Jean Charcot*, St. 20.MO.67, Strait of Scarpanto (Karpathos), Aegean Sea, 35^o^55.60’ N, 27^o^29.01’ E, 60–80m, 29.viii.1967.

2. *Scrupocellaria delilii* (Audouin, 1826)

*Crisia Delilii* Audouin, 1826: 242 [48]; Savigny, 1817: pl. 12, figs. 3.1–3.5 [49]. *Type locality*: Egypt (Red Sea). *Type material*: Presumably lost.

3. *Scrupocellaria harmeri* Osburn, 1947

*Scrupocellaria harmeri* Osburn, 1947: 20, pl. 3, figs. 1–1 [40]. *Type locality*: Aruba Island. *Holotype*: SBMNH 95952, slide, Allan Hancock Expedition, R/V *Velero III*, St. A18-39, Aruba Island, Nicholaas Bay, 12^o^21’28”N, 70^o^4’45”W, 10.iv.1939. *Paratype*: SBMNH 95953, slide, same data as holotype.

4. *Scrupocellaria incurvata* Waters, 1897

*Scrupocellaria incurvata* Waters, 1897: 9, pl. 1, figs. 16–17 [38]. *Type locality*: Mediterranean (Naples). *Syntype*: Deposited at the MM (not examined).

*Scrupocellaria aquitanica* Jullien & Calvet, 1903: 35, pl. 3, figs. 2a–b [50]. *Type locality*: Bay of Biscay. *Syntypes*: MOM 420193, two dry colonies (n.21 and n.84), *Hirondelle*, St. 58, Golfe de Gascogne, 134m, 7.viii.1886.

5. *Scrupocellaria inermis* Norman, 1867

*Scrupocellaria inermis* Norman, 1867: 203 [51]. *Type locality*: United Kingdom (Scotland). *Syntypes*: NHMUK 1911.10.1.367, dry, United Kingdom; NHMUK 1912.12.21.834, slide, United Kingdom; 1912.12.21.8334, wet, United Kingdom.

*Scrupocellaria grimaldii* Jullien & Calvet, 1903: 34, pl. 3, figs. 1a–b [50]. *Type locality*: Bay of Biscay. *Holotype*: MNHN 6064, wet, Calvet coll. N.289, Campagnes Scientifiques du Principe de Monaco, *Hirondelle*, St. 42, Golf de Gascogne, 132m, 1886.

6. *Scrupocellaria intermedia* Norman, 1893

*Scrupocellaria intermedia* Norman, 1893: 451, pl. 19, figs. 9–10 [52]. *Type locality*: Norway (Trodjhem Fjord). *Syntypes*: NHMUK 1911.10.1.369, slide; NHMUK 1912.12.21.835, wet, Trodjhem Fjord, Norway.

7. *Scrupocellaria jullieni* Hayward, 1978

*Scrupocellaria jullieni* Hayward, 1978: 208, figs. 2a–e [53]. *Type locality*: Spain. *Holotype*: MNHN 7916, wet, *Thalassa,* St. 39, Z438, 26.x.1973, 48^o^33.7’N, 10^o^25’W, 1400m.

8. *Scrupocellaria minuta* Kirkpatrick, 1888

*Scrupocellaria minuta* Kirkpatrick, 1888: 73, pl. 9, fig. 3 [54]. *Type locality*: Mauritian. *Lectotype*: NHMUK 1888.1.25.2A, slide, Mauritius. Remarks. Hayward [55] selected the lectotype for *Sl. minuta* and assigned the paralectotype specimen (NHMUK 1888.1.25.2B) to *Scrupocellaria maderensis* Busk, 1860. Examination of paralectotype, however, has revealed this specimen as an undescribed species.

9. *Scrupocellaria puelcha* (d’Orbigny, 1841) n. stat.

*Bicellaria puelcha* d’Orbigny, 1841: 8, pl. 1, figs. 9–15 [56]. *Type locality*: Argentina. *Syntype*: MNHN F.R64248, dry, d’Orbigny Collection n.13612, Patagonia, Argentina. Remarks. Lopez Gappa [57] gave differences between *Sl. scruposa puelcha* from Argentina and the specimens of *Sl. scruposa* from United Kingdom.

10. *Scrupocellaria scrupea* Busk, 1851

*Scrupocellaria scrupea* Busk, 1851: 83, pl. 9, figs. 11–12 [58]. *Type locality*: United Kingdom. *Holotype*: NHMUK 1854.11.15.79, slide (mounted by A.B. Hastings in 24.x.1927), South West coast.

11. *Scrupocellaria scruposa* (Linnaeus, 1758)

*Sertullaria scruposa* Linnaeus, 1758: 815 [59]; Ellis, 1755: 38, n. 4, pl. 20 fig. C [60]. *Type locality*: Ocean (supposedly United Kingdom). *Type material*: Presumably lost.

12. ?*Scrupocellaria macandrei* Busk, 1852

*Scrupocellaria macandrei* Busk, 1852: 24, pl. 24, figs. 1–3 [61]. *Type locality*: Spain (Mediterranean). *Holotype*: NHMUK 1854.11.14.78, slide, British Museum Catalogue Collection, Spain. Remarks. Badly preserved specimens; the morphological characteristics of the type specimen are not recognized.
