# Supplementary material for: Evidence for Polyphyly of the Genus Scrupocellaria (Bryozoa: Candidae) Based on a Phylogenetic Analysis of Morphological Characters
Source: PLoS One. 2014 Apr 18;9(4):e95296. doi: 10.1371/journal.pone.0095296 (PMC3991637; doi:10.1371/journal.pone.0095296)
Supplement: Text S4 — List of type material of Aquiloniella species. (DOCX) [file pone.0095296.s005.docx]

**Evidence for polyphyly of the genus *Scrupocellaria* (Bryozoa: Candidae) based on a phylogenetic analysis of morphological characters**

**Leandro M. Vieira^1^*, Mary E. Spencer Jones^2^, Judith E. Winston^3^, Alvaro E. Migotto^1^, Antonio C. Marques^4^**

**1** Centro de Biologia Marinha, Universidade de São Paulo, São Sebastião, SP, Brazil, **2** Department of Life Sciences, Natural History Museum, London, UK, **3** Virginia Museum of Natural History, Martinsville, VA, USA, **4** Departamento de Zoologia, Instituto de Biociências, Universidade de São Paulo, SP, Brazil

*Correspondent author. Email: leandromanzoni@hotmail.com

**Supporting Information Text S4 - List of type material of *Aquiloniella* n. gen.**

1. *Aquiloniella americana* (Packard, 1863) n. comb.

*Scrupocellaria americana* Packard, 1863: 273 [62]. *Type locality*: Labrador. *Syntype*: MCZ 134, wet, *Scrupocellaria americana*, A.S. Packard det., no locality labelled. Remarks. In the MCZ collection four other jars of supposed syntypes were found, but did not contain *Scrupocellaria* specimens: MCZ 515, *Scrupocellaria* n. sp., A.S. Packard det., Labrador; MCZ 546, MCZ 569, *Scrupocellaria americana*, A.S. Packard det., no locality labelled; MCZ 640, *Scrupocellaria americana*, A.S. Packard det., Labrador.

2. *Aquiloniella aviculareae* (Yanagi & Okada, 1918) n. comb.

*Scrupocellaria aviculareae* Yanagi & Okada, 1918: 413, pl. 4, fig. 4, text-fig. 4 [63]. *Type locality*: Japan. *Type material*: Not located.

3. *Aquiloniella orientalis* (Kluge, 1955) n. comb.

*Scrupocellaria scabra* var. *paenulata* forma *orientalis* Kluge, 1955: 106, pl. 22, fig. 4a–c [64]. *Type locality*: North Sea. *Type material*: Not located.

4. *Aquiloniella paenulata* (Norman, 1903) n. comb.

*Scrupocellaria scabra* var. *paenulata* Norman, 1903: 579 [65]. *Type locality*: Norway (Finnmark). *Type material*: Not located.

5. *Aquiloniella scabra* (van Beneden, 1848) n. comb.

*Cellarina scabra* van Beneden, 1848: 73, figs. 3–4 [66]. *Type locality*: North Sea. *Type material*: Not located.
