# Supplementary material for: Evidence for Polyphyly of the Genus Scrupocellaria (Bryozoa: Candidae) Based on a Phylogenetic Analysis of Morphological Characters
Source: PLoS One. 2014 Apr 18;9(4):e95296. doi: 10.1371/journal.pone.0095296 (PMC3991637; doi:10.1371/journal.pone.0095296)
Supplement: Text S5 — List of type material of Aspiscellaria n. gen. (DOCX) [file pone.0095296.s006.docx]

**Evidence for polyphyly of the genus *Scrupocellaria* (Bryozoa: Candidae) based on a phylogenetic analysis of morphological characters**

**Leandro M. Vieira^1^*, Mary E. Spencer Jones^2^, Judith E. Winston^3^, Alvaro E. Migotto^1^, Antonio C. Marques^4^**

**1** Centro de Biologia Marinha, Universidade de São Paulo, São Sebastião, SP, Brazil, **2** Department of Life Sciences, Natural History Museum, London, UK, **3** Virginia Museum of Natural History, Martinsville, VA, USA, **4** Departamento de Zoologia, Instituto de Biociências, Universidade de São Paulo, SP, Brazil

*Correspondent author. Email: leandromanzoni@hotmail.com

**Supporting Information Text S5 - List of type material of *Aspiscellaria* n. gen.**

1. *Aspiscellaria bellula* (Osburn, 1947) n. comb.

*Scrupocellaria bellula* Osburn, 1947: 21, pl. 4, figs. 1–3 [40]. *Type locality*: Aruba Island, Caribbean. *Holotype*: SBMNH 95954, slide, AHF 4, St. A18039, At 505, Allan Hancock Expedition, R/V *Velero III*, St. A18-39, Atlantic, Aruba Island, San Nicholaas Bay, 12°21’28” N, 70°4’45” W, 42m (23fms), 10.iv.1939. *Paratype*: SBMNH 95955, slide, same data as holotype.

2. *Aspiscellaria carmabi* (Fransen, 1986) n. comb.

*Scrupocellaria carmabi* Fransen, 1986: 12, figs. 16a–d [68]. *Type locality*: Curaçao. *Holotype*: RMNH 02977, slide, St. Cur82.051, Netherlands Antilles, Curaçao, Spaanse water, Inner Bay, near Jan Sofat. 0–1m, 19.viii.1982. *Paratypes*: RMNH 02977 (see remarks), wet, same data as holotype; RMNH 03042, wet, St. Cur82.057, Netherlands Antilles, Curaçao, Piscadera Inner Bay, entrance, on slope of canal (recently dug), 2–6m, 5.ix.1982; RMNH 03043, wet, St. Cur82.077, Netherlands Antilles, Curaçao, Spaanse water, entrance, east shore of Spaanse Lagoen, 0–1m, 17.ix.1982; RMNH 03044, wet, St. Cur82.029, Netherlands Antilles, Curaçao, Spaanse water, Inner Bay, Brakke Put, 0–1m, 9.viii.1982; RMNH 03045, wet, St. Cur82.078, Netherlands Antilles, Curaçao, Spaanse water, Inner Bay, near New Haven, 0–1m, 17.ix.1982; RMNH 0346, wet, St. Cur82.031, Netherlands Antilles, Curaçao, Fuikbaai, eastern part, 0–1m, 9.viii.1982; RMNH 0347, wet, St. Cur82.061, Netherlands Antilles, Curaçao, Fuikbaai, western part, 3–6m, 8.ix.1982; RMNH 0348, wet, St. Cur82.066, Netherlands Antilles, Curaçao, Fuikbaai, eastern part, 0–1m, 10.ix.1982; RMNH 0349, slide, St. PWH.1493, Netherlands Antilles, Curaçao, Piscadera Baai, Northern part, NW inlet of Piscadera Chikitu, 0–1m, 25.xi.1963; RMNH 0350, slide, St. PWH.1629, Netherlands Antilles, Curaçao, Spaanse water, inner bay, Jan Sofat, islet, 0–1m, 17.xi.1968; RMNH 0351, wet and slide, St. PWH.1039, Netherlands Antilles, Curaçao, Fuikbaai, W part, SE of Newport Bath, 0–1.5m, 20.xi.1948; RMNH 0352, wet and slide, St. PWH.1039, Netherlands Antilles, Curaçao, Fuikbaai, W part, SE of Newport Bath, 0–2m, 20.xi.1948. Remarks. Few colonies stored in alcohol under number RMNH 03042 were also labelled as holotype. However, the holotype designed in original description is the figured specimens stored as one balsam slide [68] and the additional specimens in alcohol are paratypes.

3. *Aspiscellaria cornigera* (Pourtalès, 1867) n. comb.

*Canda cornigera* Pourtalès, 1867: 110 [69]. *Type locality*: Florida, USA. *Type material*: Presumably lost.

4. *Aspiscellaria frondis* (Kirkpatrick, 1890) n. comb.

*Scrupocellaria frondis* Kirkpatrick, 1890: 504, text-fig. 1 [70]. *Type locality*: Brazil (Pernambuco). *Holotype*: NHMUK 1888.4.16.20, dry, Pernambuco, Brazil.

5. *Aspiscellaria hildae* (Fransen, 1986) n. comb.

*Scrupocellaria hildae* Fransen, 1986: 54, figs. 18a–d [68]. *Type locality*: Curaçao. *Holotype*: RMNH 02980, slide, St. Cur82.044, Netherlands Antilles, Curaçao, St Marta Bay entrance, 0.3–2m, 16.viii.1982. *Paratypes*: RMNH 02980 (see remarks), wet, same data as holotype; RMNH 03039, wet and slides, St. Cur82.002, Netherlands Antilles, Curaçao, Piscadera Outer Bay, under Hilton pier, 3–6 m, 4.viii, 5.ix, and 11.ix.1982; RMNH 03065, wet, St. Cur82.027, Netherlands Antilles, Curaçao, Spaanse water, entrance, near Punta Cabajero, 1.5–3m, 9.viii.1982; RMNH 03066, wet, St. Cur82.077, Netherlands Antilles, Curaçao, Spaanse water, entrance, east shore of Spaanse Lagoen, 0–1m, 17.ix.1982; RMNH 03067, wet, St. Cur82.033, Netherlands Antilles, Curaçao, Fuikbaai, eastern part, 1.5–3m, 9.viii.1982. RMNH 03068, wet, St. Cur82.024, Netherlands Antilles, Curaçao, St. Michiel Outer Bay, 2m, 7.viii.1982; RMNH 03069, slide, St. PWH.1039, Netherlands Antilles, Curaçao, Fuikbaai, W part, SE of Newport Bath, 0–1.5m, 20.xi.1948; RMNH 03070, slide, St PWH.1068a, Netherlands Antilles, Bonaire, Lac, entrance, Boca behind reef, 1–2m, x.1930. Remarks. A few colonies stored in alcohol under number 02980 were also labelled as holotype. The holotype designed in original description, however, is the species on one balsam slide [68] and the additional specimens in alcohol are paratypes.

6. *Aspiscellaria panamensis* (Osburn, 1950) n. comb.

*Scrupocellaria panamensis* Osburn, 1950: 141, pl. 17, figs. 5–6, pl. 20, fig. 2 [12]. *Type locality*: Panama. *Holotype*: SBMNH 96158, slide, Las Perlas Island, Panama, 8^o^22’N, 79^o^1’60”W. *Paratypes*: SBMNH 96159–60, slides, AHF 470, Charles Island, Galapagos, 0.5 mile north of Black Beach, 1^o^16’46”S, 90^o^29’56”W, 16.4m (9fms), 14.12.1934; USNM (uncatalogued), slide, AHF 850-38, off Cape San Francisco, Ecuador, 27.3m (15fms).

7. *Aspiscellaria piscaderaensis* (Fransen, 1986) n. comb.

*Scrupocellaria piscaderaensis* Fransen, 1986: 51, figs. 17a–b [68]. *Type locality*: Curaçao. *Holotype*: RMNH 02979, slide, St. Cur82.043, Netherlands Antilles, Curaçao, Piscadera Inner Bay, southern part, Candelchi, 0–0.5m, 14.viii.1982. *Paratypes*: RMNH 02979 (see remarks), wet, same data as holotype; RMNH 03053, wet and slide, St. Cur82.006, Netherlands Antilles, Curaçao, Piscadera Inner Bay, southern part, north of Marie Pampoen, 0–0.5m, 4.viii.1982; RMNH 03055, wet, St. Cur82.034a, Netherlands Antilles, Curaçao, Piscadera Inner Bay, southern part, near Punta Kibracos, 0.8–2m, 11.viii.1982; RMNH 03056, wet, St. Cur82.035, Netherlands Antilles, Curaçao, Piscadera Inner Bay, southern part, near Punta Kibracos, 0–0.8m, 11.viii.1982; RMNH 03057, wet, St. Cur82.046, Netherlands Antilles, Curaçao, Piscadera Inner Bay, southern part, near Candelchi, 0–0.5m, 17.viii.1982; RMNH 03058, wet and slide, St. Cur82.046a, Netherlands Antilles, Curaçao, Piscadera Inner Bay, southern part, near Candelchi, 0.4–0.8m, 17.viii.1982; RMNH 03059, wet, St. Cur82.047, Netherlands Antilles, Curaçao, Piscadera Inner Bay, southern part, near Candelchi, 0–1m, 17.viii.1982; RMNH 03060, wet and slide, St. Cur82.048, Netherlands Antilles, Curaçao, Piscadera Inner Bay, southern part, near Candelchi, 0–1m, 17.viii.1982; RMNH 03061, wet, St. Cur82.049, Netherlands Antilles, Curaçao, Piscadera Inner Bay, southern part, western shore, 0–0.08m, 18.viii.1982; RMNH 03062, wet and slide, St. Cur82.036, Netherlands Antilles, Curaçao, Piscadera Inner Bay, central part, near Punta Kibracos, 0–0.5m, 12.viii.1982. Remarks. A few colonies stored in alcohol under number 02979 were also labelled as holotype. The holotype designed in original description, however, is the balsam slide with the figured specimens [68] and the additional specimens in alcohol are paratypes.

8. *Aspiscellaria unicornis* (Liu, 1980) n. comb.

*Scrupocellaria unicornis* Liu, 1980: 179, figs. 1–2 [71]. *Type locality*: China. *Type material*: Not located.
