# Supplementary material for: Evidence for Polyphyly of the Genus Scrupocellaria (Bryozoa: Candidae) Based on a Phylogenetic Analysis of Morphological Characters
Source: PLoS One. 2014 Apr 18;9(4):e95296. doi: 10.1371/journal.pone.0095296 (PMC3991637; doi:10.1371/journal.pone.0095296)
Supplement: Text S6 — List of type material of Paralicornia n. gen. (DOCX) [file pone.0095296.s007.docx]

**Evidence for polyphyly of the genus *Scrupocellaria* (Bryozoa: Candidae) based on a phylogenetic analysis of morphological characters**

**Leandro M. Vieira^1^*, Mary E. Spencer Jones^2^, Judith E. Winston^3^, Alvaro E. Migotto^1^, Antonio C. Marques^4^**

**1** Centro de Biologia Marinha, Universidade de São Paulo, São Sebastião, SP, Brazil, **2** Department of Life Sciences, Natural History Museum, London, UK, **3** Virginia Museum of Natural History, Martinsville, VA, USA, **4** Departamento de Zoologia, Instituto de Biociências, Universidade de São Paulo, SP, Brazil

*Correspondent author. Email: leandromanzoni@hotmail.com

**Supporting Information Text S6 - List of type material of *Paralicornia* n. gen.**

1. *Paralicornia* *hamata* (Tilbrook & Vieira, 2012) n. comb.

*Scrupocellaria hamata* Tilbrook & Vieira, 2012: 42, fig. 9 [11]. *Type locality*: Australia (Queensland). *Holotype*: MTQ G25166, HI10–055, Channel between Heron Island and Wistari Reef, Great Barrier Reef, 23.44982° S, 151.91255° E, 26 November 2010, 19 m, coll. KJT.

2. *Paralicornia* *limatula* (Hayward, 1988) n. comb.

*Scrupocellaria limatula* Hayward, 1988: 286, figs. 3d–e [55]. *Type locality*: Mauritius. *Holotype*: NHMUK 1987.1.18.41, wet, Tamarin, Mauritius, close to reef crest.

3. *Paralicornia* *obtecta* (Haswell, 1880) n. comb.

*Scrupocellaria obtecta* Haswell, 1880: 37 [72]. *Type locality*: Australia (Queensland). *Syntype*: NHMUK 1928.9.13.103, dry, W.A. Haswell, Port Denison, Queensland, Australian Museum, Reg. Oct. 23, 1899.

4. *Paralicornia* *pusilla* (Smitt, 1872) n. comb.

*Cellularia pusilla* Smitt, 1872: 13, pl. 5, figs. 32–34 [73]. *Type locality*: off Tortugas. *Syntype*: MCZ 0100, dry, Cast No. 13, Pourtàles, W off Tortugas, 124m (68fms) [74].

5. *Paralicornia* *sinuosa* (Canu & Bassler, 1927) n. comb.

*Scrupocellaria sinuosa* Canu & Bassler, 1927: 4, pl. 1, figs 4–5 [75]. *Type locality*: Hawaii. *Holotype*: USNM 8426, *Albatross* St. 3999, Pacific II, vicinity of Kawai Island, Hawaii.

6. *Paralicornia* *spatulatoidea* (Liu, 1980) n. comb.

*Scrupocellaria spatulatoidea* Liu, 1980: 180, figs. 3–8 [71]. *Type locality*: China. *Type material*: Not located.

7. *Paralicornia* *unguiculata* (Osburn, 1950) n. comb.

*Scrupocellaria unguiculata* Osburn, 1950: 148, pl. 17, figs. 1–2, pl. 19, fig. 6 [12]. *Type locality*: Galapagos. *Holotype*: SBMNH 96169, slide, Allan Hancock Expedition, R/V *Velero III*, St. 795-38, Sulivan Bay, James Island, Galapagos, 0^o^16’12”S, 90^o^34’50”W, 65.8–73.1m (36–40fms), 20.i.1938. *Paratypes*: SBMNH 96170, slide, AHF 450, Albemarle (=Isabela), Galapagos, 0^o^55’00”S, 90^o^30’00”W, 109.7m (60fms), 26.i.1934; SBMNH 96171, slide, AHF 451, Post Office Bay, Charles Island, Galapagos, 1^o^11’15”S, 90^o^31’10”W, 182.9m (100fms), 29.i.1934.

8. *Paralicornia spatulata* (d’Orbigny, 1851) *species inquirenda*

*Cellularia spatulata* d’Orbigny, 1851: 50 [2] (referred to Savigny, 1817, pl. 12, fig 1 [49]). *Type locality*: Egypt (Mediterranean or Red Sea?). *Type material*: Presumably lost.
