# Supplementary material for: Evidence for Polyphyly of the Genus Scrupocellaria (Bryozoa: Candidae) Based on a Phylogenetic Analysis of Morphological Characters
Source: PLoS One. 2014 Apr 18;9(4):e95296. doi: 10.1371/journal.pone.0095296 (PMC3991637; doi:10.1371/journal.pone.0095296)
Supplement: Text S7 — List of type material of Pomocellaria n. gen. (DOCX) [file pone.0095296.s008.docx]

**Evidence for polyphyly of the genus *Scrupocellaria* (Bryozoa: Candidae) based on a phylogenetic analysis of morphological characters**

**Leandro M. Vieira^1^*, Mary E. Spencer Jones^2^, Judith E. Winston^3^, Alvaro E. Migotto^1^, Antonio C. Marques^4^**

**1** Centro de Biologia Marinha, Universidade de São Paulo, São Sebastião, SP, Brazil, **2** Department of Life Sciences, Natural History Museum, London, UK, **3** Virginia Museum of Natural History, Martinsville, VA, USA, **4** Departamento de Zoologia, Instituto de Biociências, Universidade de São Paulo, SP, Brazil

*Correspondent author. Email: leandromanzoni@hotmail.com

**Supporting Information Text S7 - List of type material of *Pomocellaria* n. gen.**

1. *Pomocellaria* *californica* (Trask, 1857) n. comb.

*Scrupocellaria californica* Trask, 1857: 114, pl. 4 fig. 2 [76]. *Type locality*: Bay of San Francisco, California. *Type material*: Presumably lost.

*Scrupocellaria brevisetis* Hincks, 1882: 462 [77]. *Type locality*: Queen Charlotte Island, British Columbia. *Type material*: No record at NHMUK, presumably lost. Remarks. Robertson [78] suggested *Scrupocellaria brevisetis* Hincks, 1882 was a junior synonym of *Scrupocellaria californica* Trask, 1857.

2. *Pomocellaria* *inarmata* (O’Donoghue & O’Donoghue, 1926) n. comb.

*Scrupocellaria inarmata* O’Donoghue & O’Donoghue, 1926: 41 [79]. *Type locality*: British Columbia (Tricomali Channel). *Holotype*: NHMUK 1964.4.2.10, dry, Pacific coast of N. America.

3. *Pomocellaria* *talonis* (Osburn, 1950) n. comb.

*Scrupocellaria talonis* Osburn, 1950: 147, pl. 17, fig. 3; pl. 19, fig. 3; pl. 20, fig. 7 [12]. *Type locality*: Panama. *Holotype*: SBMNH 96168, AHF 39 (ex. Osburn Collection), Perlas Island, Panama, 8^o^22’0”N, 79^o^1’60”W.

4. *Pomocellaria* *varians* (Hincks, 1882) n. comb.

*Scrupocellaria varians* Hincks, 1882: 461, pl. 19, figs 1–1c [77]. *Type locality*: Queen Charlotte Island, British Columbia. *Holotype*: NHMUK 1886.3.6.12, slide (mounted by A.B. Hastings 25.10.1927), Queen Charlotte Island.
