# Supplementary material for: Evidence for Polyphyly of the Genus Scrupocellaria (Bryozoa: Candidae) Based on a Phylogenetic Analysis of Morphological Characters
Source: PLoS One. 2014 Apr 18;9(4):e95296. doi: 10.1371/journal.pone.0095296 (PMC3991637; doi:10.1371/journal.pone.0095296)
Supplement: Text S8 — List of type material of Scrupocaberea n. gen. (DOCX) [file pone.0095296.s009.docx]

**Evidence for polyphyly of the genus *Scrupocellaria* (Bryozoa: Candidae) based on a phylogenetic analysis of morphological characters**

**Leandro M. Vieira^1^*, Mary E. Spencer Jones^2^, Judith E. Winston^3^, Alvaro E. Migotto^1^, Antonio C. Marques^4^**

**1** Centro de Biologia Marinha, Universidade de São Paulo, São Sebastião, SP, Brazil, **2** Department of Life Sciences, Natural History Museum, London, UK, **3** Virginia Museum of Natural History, Martinsville, VA, USA, **4** Departamento de Zoologia, Instituto de Biociências, Universidade de São Paulo, SP, Brazil

*Correspondent author. Email: leandromanzoni@hotmail.com

**Supporting Information Text S8 - List of type material of *Scrupocaberea* n. gen.**

1. *Scrupocaberea dongolensis* (Waters, 1909) n. comb.

*Scrupocellaria scrupea* var. *dongolensis* Waters, 1909: 134 [80]. *Type locality*: Indian waters. *Syntypes*: NHMUK 1928.9.13.98, dry, Thornely coll., Mannar, Ceylon (Sri Lanka), Rep. Pearl, Oyster Fisheries, XXVI, 1905, p. 109. Miss. L.R. Thornely, Reg. Apr. 25, 1906; NHMUK 1899.7.1.804 and 806, dry, G. Busk coll., Trincomalee, Sri Lanka, G. Johnston.

2. *Scrupocaberea gilbertensis* (Maplestone, 1909) n. comb.

*Scrupocellaria gilbertensis* Maplestone, 1909: 411, pl. 26, fig. 2 [81]. *Type locality*: Gilbert Island. *Holotype*: MV 45061, slide, Gilbert Island.

3. *Scrupocaberea maderensis* (Busk, 1860) n. comb.

*Scrupocellaria maderensis* Busk, 1860: 280 [82]. *Type locality*: Madeira. *Holotype*: NHMUK 1899.7.1.780, dry, G. Busk coll., Madeira.

4. *Scrupocaberea ornithorhynchus* (Thomson, 1858) n. comb.

*Scrupocellaria ornithorhynchus* Thomson, 1858: 90 [83]. *Type locality*: Australia (Bass Strait). *Holotype*: NHMUK 1899.7.1.783, dry, G. Busk coll., Australia.
